# Detection of genomic structure variants associated with wrinkled skin in Xiang pig by next generation sequencing

**DOI:** 10.18632/aging.203711

**Published:** 2021-11-27

**Authors:** Liu Xiaoli, Hu Fengbin, Huang Shihui, Niu Xi, Li Sheng, Wang Zhou, Ran Xueqin, Wang Jiafu

**Affiliations:** 1 Institute of Agro-Bioengineering, Key Laboratory of Plant Resource Conservative and Germplasm Innovation in Mountainous Region (Ministry of Education), College of Life Science and College of Animal Science, Guizhou University, Guiyang 550025, China

**Keywords:** Xiang pig, skin wrinkles, aging, structural variation

## Abstract

Wrinkling is prominent manifestation of aging skin. A mutant phenotype characterized by systemic wrinkles and thickened skin was discovered in Xiang pig populations with incidence about 1-3%. The feature in histological structure was epidermal hyperplasia and thickening, collagen fibers disorder. To uncover genetic mechanisms for the mutant phenotype of Xiang pigs with systemic wrinkle (WXP), a genome-wide of structural variations (SVs) in WXP was described by next generation resequencing, taking Xiang pigs (XP) and European pigs (EUP) as compares. Total of 32,308 SVs were detected from three pig groups and 965 SVs were identified specifically from WXP, involving 481 protein-coding genes. These genes were mainly enriched in nuclear structure, ECM components and immunomodulatory pathways. According to gene function and enrichment analysis, we found that 65 candidate SVs in 59 protein genes were probably related with the systemic wrinkle of WXP. Of these, several genes are reported to be associate with aging, such as *EIF4G2*, *NOLC1*, *XYLT1*, *FUT8, MDM2* and so on. The insertion/deletion and duplication variations of SVs in these genes resulted in the loss of stop-codon or frameshift mutation, and aberrant alternative splicing of transcripts. These genes are involved in cell lamin filament, intermediate filament cytoskeleton, supramolecular complex, cell differentiation and regulation of macromolecule metabolic process etc. Our study suggested that the loss of function or aberrant expression of these genes lead to structural disorder of nuclear and the extracellular matrix (ECM) in skin cells, which probably was the genetic mechanisms for the mutant phenotype of systemic skin wrinkle of Xiang pig.

## INTRODUCTION

Skin is a complex organ covering the entire surface of animal body. The skin aging processes are affected by intrinsic or extrinsic factors [1]. One of clinical manifestation of skin aging is wrinkles [2]. Whether caused by internal or external factors, wrinkles are inseparable from cutaneous aging. In the action of intrinsic factors, the proliferation of cells is reduced, and the senescence process of cells are increased, resulting in structural and physiological disorders, and forming a thin and atrophic, finely wrinkled skin [1]. Under the stimulation of external factors (ultraviolet rays), the reactive oxygen species (ROS) accumulate, causing cell proliferation impaired, metalloproteinase expression increased, and dermal extracellular matrix degraded, then taking deep and laxity wrinkles [3, 4].

Over past decades, research has gained crucial insight into underlying genetic factors of skin aging, especially the contribution of genetic variation. Such as single nucleotide polymorphisms. The mutation c.1824C > T of Lamin A/C gene (*LMNA*) is confirmed to be the cause of Hutchinson-Gilford Progeria Syndrome (HGPS) [5]. The rs185146, rs12203592, and rs4268748 polymorphisms near *SLC45A2*, *IRF4*, and *MC1R*, respectively, are associated with pigmentation and skin aging [6]. The insertion/deletion (InDel) variations are also related with skin aging. A recessive c.1030_1033delCTGT deletion in *FOXN1* caused skin wrinkles and hairlessness in Barman cats [7]. Similarly, the gain or loss of copy number may also be involved in skin aging. The gaining copy number of the *HAS2* gene is associated with a strong selection for wrinkles phenotypes in the Chinese Shar-Pei dogs [8]. Importantly, the contribution of structural variations (SVs) to complex phenotypes accounted for 83.6% percent of the total genetic variation [9]. For example, a translocation or a deletion that removes the C-terminus of *ELN* gene can cause cutis laxa [10]. Large scale deletion of mitochondrial genome leads to wrinkles phenotypes, such as the 4,977 bp common deletion [11]. In addition, gene expression, microRNA regulation and epigenetic factors also play an important role in skin aging [12–14]. Skin aging seems to involve multiple genes.

The Xiang pig is a miniature pig breed that originated in the mountainous area of Guizhou Province in China. Some Chinese indigenous pigs have obvious wrinkles on the forehead, such as Chinese Erhualian and Meishan. It’s reported that the G allele in *GRM4* is beneficial to increase Erhualian pig facial wrinkles by using genome-wide association analysis [15]. Evidence of selective sweeps in the genome of Meishan pig reveals that strong selective sweep signals of NFKB1 may have resulted in the wrinkled skin and face [16]. Xiang pigs also have characteristic facial wrinkles like other native pig breeds. But a few individuals displayed dense wrinkles on the back and buttock. This is a special systemic skin wrinkle phenotype, and it can be inherited, with an incidence of about 1-3%. So far, the cause of the systemic skin wrinkles in Xiang pig is still enigmatic. In the present study, we employed histology methods to characterize the structure of the skin in Xiang pigs with skin wrinkled, and we performed whole-genome resequencing using Illumina HiSeq2500 to identify genomic structure variants in these pigs. The aim of this study is to uncover genetic variants associated with skin wrinkles of Xiang pig. It might help us to better understand the underlying molecular mechanism of skin aging, which probably was the genetic mechanisms for the mutant phenotype of Xiang pigs with systemic wrinkle.

## RESULTS

### Phenotype description

**Figure 1:**
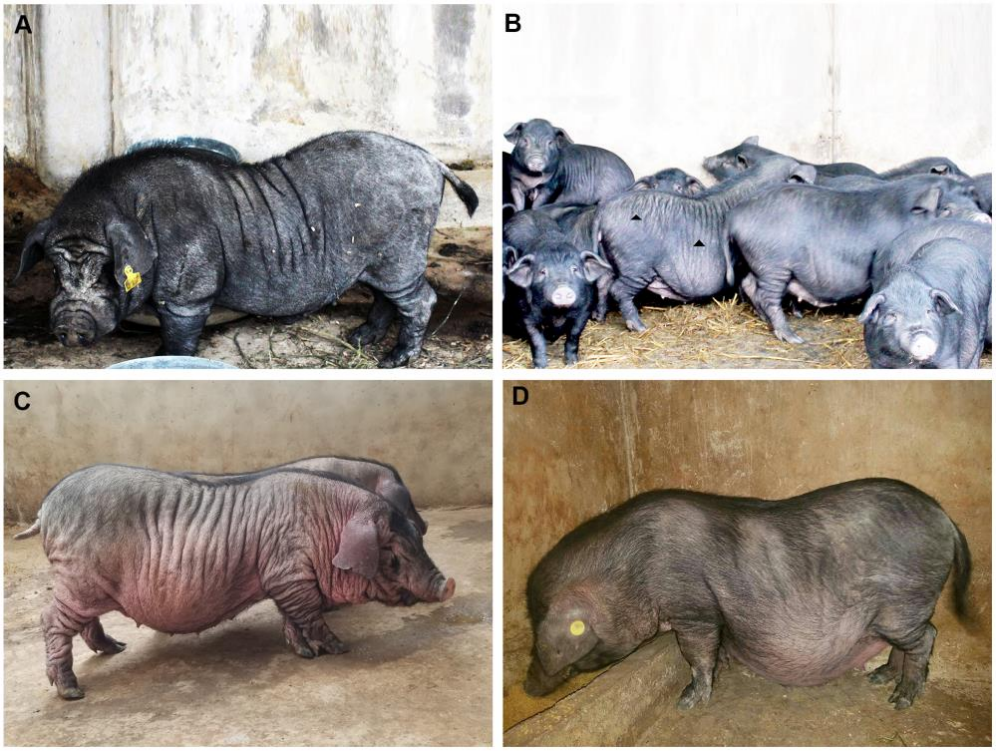
**The phenotype spectrum of Xiang pig.** Originally, one boar, except for the characteristic facial wrinkles, were only a few wrinkles on the side of the trunk and no wrinkles on the buttocks (**A**). Gradually, it spread to other individuals. There were obvious wrinkles on the trunk and hip sides (**B**). Finally, deep and wide wrinkles were seen in a few individuals (**C**), but no obvious wrinkles were seen in normal Xiang pigs (**D**).

The systemic skin wrinkle was a newly discovered phenotype in Xiang pigs. The mutated phenotype was originally observed in a boar (Figure 1A). The trait showed autosomal recessive heredity among his offspring, with an incidence of about 1-3% in the populations (Supplementary Table 1). Piglets first appeared wrinkles after two months of birth. And the degree of wrinkle was increased with age (Figure 1C). The wrinkled skin in 12-month-old pigs displayed a sparse short fur and lighter color skin. The skin of the trunk and limbs formed dense and deep wrinkles, with 0.5-2 cm in depth, 3-5 cm in width and 5-32 cm in length. However, they had no significant difference in feeding behavior, growth rate, weight, and fertility compared with normal Xiang pigs.

### Histological structure of wrinkle skin

**Figure 2:**
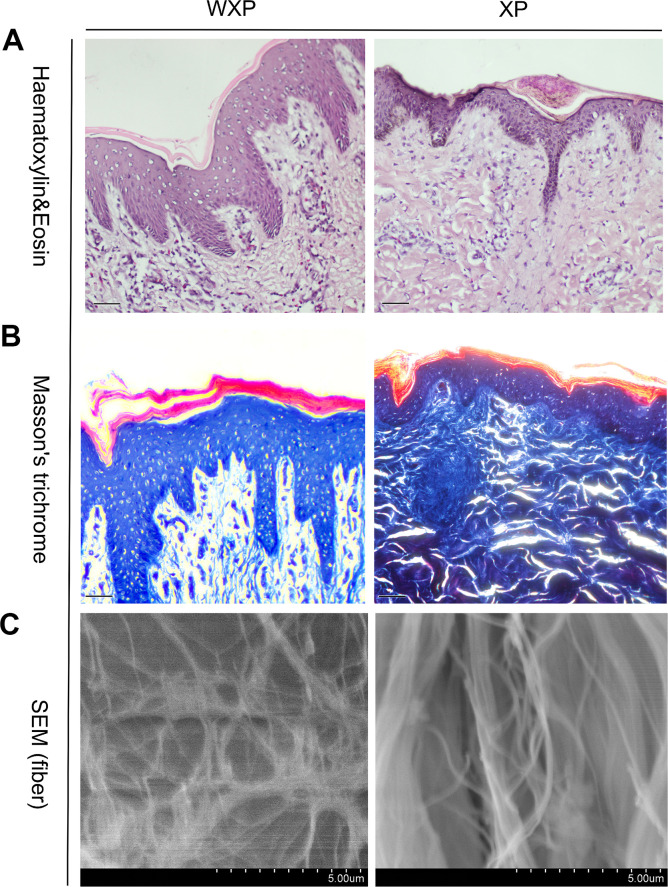
**Skin histological features abnormal in Xiang pigs with systemic wrinkle (WXP).** Representative Haematoxylin and Eosin and Masson's trichrome-stained skin sections of WXP and XP. And scanning electron microscope to observed the collagen fiber structure of the dermis. Scale bars: 50 μm in (**A**, **B**) and 5 μm in (**C**).

The skin directly above the longissimus dorsi muscle were evaluated anatomic and histological structures. It was found that the trunk skin of WXP was thickened and the subcutaneous fat layer became thinner (Supplementary Table 2), and no obvious changes was found in other internal organs. H&E-stained for wrinkled skin showed that the skin surface was uneven and the thickness was not uniform. The epidermal layer was proliferated especially in both granular and spinous layers, and projected downward into the dermis layer. The skin thickness was about twice than that of a normal Xiang pig (Supplementary Table 3, *P* < 0.05). Masson’s trichrome staining indicated that the collagen fibers were disordered and the stratification was indistinct. In addition, the collagen bundles in dermis decreased and became loose under scanning electron microscopy (Figure 2).

### Detection of genomic structural variation

The Xiang pig genomes from wrinkle and normal samples were sequenced using Illumina HiSeq2500 and all data were subjected to quality assessment to obtain clean data. In addition, we downloaded the genome resequencing data of 21 European pigs (EUP) belonging to three breeds from NCBI database, and followed the same method for quality assessment. Total of 0.95 Tb sequences with an average depth of 9.90 × were obtained form 35 pigs (Table 1 and Supplementary Table 4).

**Table 1:** Summary of sequencing and mapping statistics.

**Sample**	**Raw base(G)**	**Clean base(G)**	**Map base(G)**	**Map ratio(%)**	**Depth(X)**	**Q20(%)**	**GC (%)**
WXP1	32.26	27.54	26.34	95.64	10.77	93.20	44.99
WXP2	32.08	27.00	25.73	95.30	10.52	92.80	45.14
WXP3	30.39	22.37	21.65	96.78	8.85	89.13	44.92
WXP4	30.16	22.46	21.54	95.90	8.81	89.44	43.39
WXP5	33.22	30.88	29.85	96.66	12.21	97.38	42.30
WXP6	35.18	34.01	32.83	96.53	13.43	97.42	42.46
WXP7	35.15	33.98	32.83	96.62	13.43	97.49	42.55
XP1	30.11	28.31	27.42	96.86	11.21	96.30	42.52
XP2	33.52	31.69	30.63	96.66	12.53	96.50	42.26
XP3	48.46	42.25	39.82	94.25	16.29	92.90	42.42
XP4	29.22	27.41	26.56	96.90	10.86	96.50	42.64
XP5	30.88	29.20	28.22	96.64	11.54	96.19	42.94
XP6	48.62	42.68	40.23	94.26	16.45	92.75	41.73
XP7	35.23	34.05	32.88	96.56	13.45	97.38	42.43

**Figure 3:**
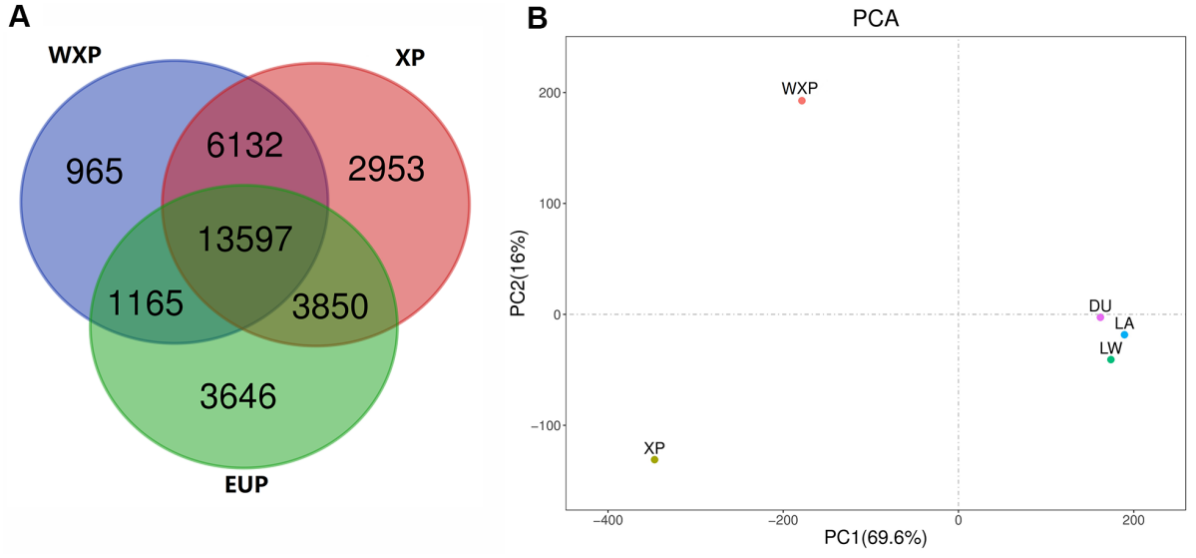
**Venn diagram showing the overlap of identified SVs, and principal component analyses for all of three pig groups.** (**A**) Venn diagram showing the overlap of identified SVs in the WXP, XP and EUP groups. (**B**) PCA plot with SVs data. Different colors represent different subspecies.

Then, both softwares of Pindel and SoftSV were used to call SVs, with 488,269 SVs by Pindel and 667,276 SVs by SoftSV (Supplementary Table 5), respectively. The SVmerge of human method were used to merge SVs between different individual to obtain non-redundant SVs, and only SVs detected by two or more individuals were retained. Finally, we detected 32,308 SVs events among the 35 individuals, named sv00001-sv32308 (Supplementary Table 6), including 27,752 deletions (DEL), 2,327 tandem duplications (DUP), 2,035 insertions (INS) and 194 inversions (INV). The 32,308 SVs covered 18.52 Mb of pig genome. The distribution of SVs was random in pig genome. We observed that chromosome 1 contains the most SVs and chromosome 18 contains the least. The largest SV (20,287 bp in size, sv_16660) was found on chromosome 9. In addition, the number, size, and coverage ratio of SVs were different in these pig breeds. Out of the identified SVs, 13,597 SVs were shared in the genomes of three groups (Figure 3A).

We carried out principal component analysis (PCA) on the SV pattern data (Figure 3B). The PC1 differentiated between China (XP AND WXP) and European pig breeds (LW, LA, DU) geographically. The PC2 reflected the biological distinguish from XP and WXP. It suggested that the data groups were clearly clustered and can be used for subsequent screening of mutation sites.

### Screening and annotation of specific SVs in Xiang pig group with wrinkle skin

**Figure 4:**
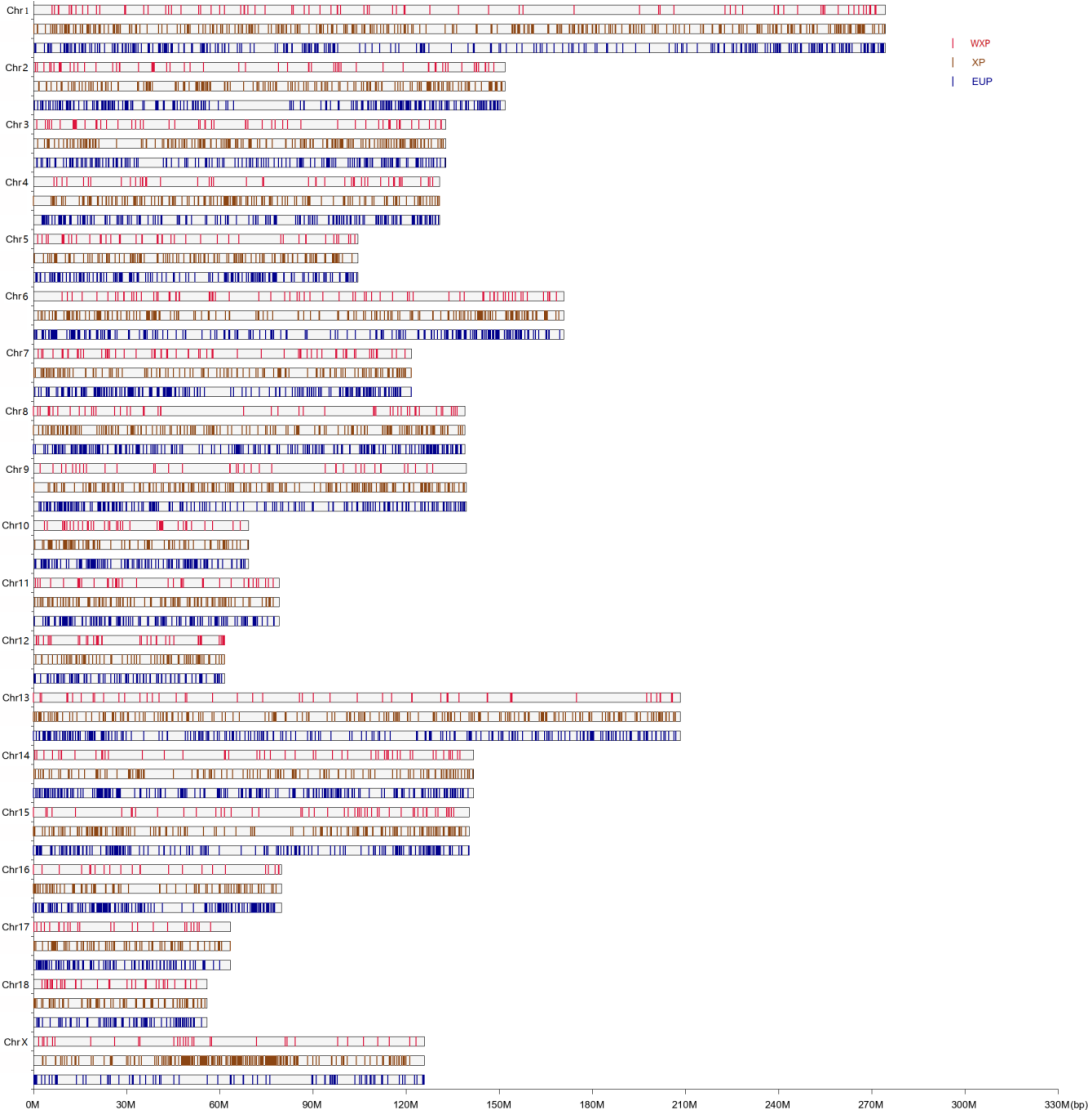
**The chromosome distribution of the group-specific in WXP, XP, and EUP pig group.** The vertical bars indicated different pig group with the crimson, saddle brown and dark blue color for WXP, XP and EUP, respectively.

**Figure 5:**
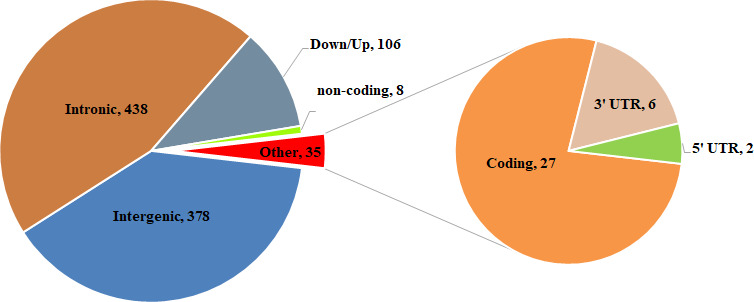
Summary of functional classification of WXP_specific SVs identified.

Compared SV distribution among three groups (Figure 3A), we identified 965 specific SVs in WXP group (DEL=715; DUP=78; INS=167; INV=5). The distribution of these population-specific SVs was presented in Figure 4 and Supplementary Table 7. Among the SV types identified, the indels (insertion and deletion) were the most abundant structure variant types (91.4%). Both DUPs and INVs only accounted for 8.6% of total SVs. It showed that these SVs were randomly distributed on all chromosomes except for chromosome Y. We also examined the genomic location of the b WXP-specific SVs and found that most of them (n = 438, 45.38%) were located in intron and that 378 SVs (39.17%) were located in intergenic regions, 35 SVs (3.62%) in exon or exon-intron regions, 106 SVs (10.98%) in upstream or downstream region of genes, 8 SVs (0.83%) in untranslated regions (Figure 5). These SVs impacted 553 Ensembl genes (Supplementary Table 7), including 481 protein-coding genes, 2 pseudogenes, and 37 noncoding RNA genes. Most of these genes (n=527, 92.3%) contained only a single SVs, while fewer of them (n=26, 4.7%) harbored two or more SVs. We further predicted the impact of SV events on host genes (Supplementary Table 7). We believed that 60 SVs have an important impact on gene function by Variant Effect Predictor (VEP) annotation. Only three of deletions were predicted to have a high impact, including stop lost on *CHD3* and novel gene (ENSSSCG00000029231*)*, frameshift variants in uncharacterized protein gene (ENSSSCG00000037023). 23 deletions/insertions and 1 duplication in exon or exon-intron regions had an important impact as modifier, including 4 frameshift variants on *RBPJ, PPP2R1A, PRRC2A*, and *TNXB* genes, 13 potential aberrant splicing variants on 12 genes (*LMNA*, *NOLC1*, *MAGED1*, *HGS*, *EIF4G2*, *DDX5*, *HSP90AB1*, *ATP5F1B*, *RAB1A, PABPC1, PABPC1* and *NEFM*). Eight SVs changed the UTRs length of 8 genes (*MDM2*, *CHD3*, *ROS*1, *ABCF3*, *HLX* and 3 novel genes). Additionally, we found that 25 deletions/insertions resulted in loss of TFs binding sites and promoters of 23 genes (including *TFF2*, *SFXN2*, *RNF14*, *RARA*, *RALGPS1*, *PRDX1*, *MTMR8, MTBP, LMNB1, EIF6, DNAJB14, COLQ, CCL5, C12orf73*).

### Gene enrichment and function annotation

In order to obtain insight into the biological functions of WXP-specific SVs harbored genes, KEGG pathway and GO enrichment was performed using the KOBAS3.0 bioinformatics resource (Supplementary Table 8). The GO analyses revealed 132 significant GO terms (*P* < 0.05), which were mainly comprised of nuclear lamina structure, RNA biosynthetic, protein catabolic, cell apoptotic, immune response, vesicular transport. Besides, these genes were also enriched in terms of nuclear lamina (*P* = 3.73 × 10^−4^). In KEGG pathway, genes affected by WXP-specific SVs mainly involved in RNA transport (ssc03013), Notch signaling pathway (ssc04330), Wnt signaling pathway (ssc04310) and so on. These pathways participate in the processes of cellular senescence, tumorigenesis, and cellular autophagy.

Ultimately, based on Gene function and enrichment analysis of WXP-specific SVs, the wrinkle related SVs were screened according to the three criteria: (1) Specific mutation was identified only from the wrinkle skin of Xiang pigs; (2) The SVs might affect genes enriched in pathways of the formation of skin structures and aging; (3) The SVs related with genes associated with aging, based on previous reports (https://www.ncbi.nlm.nih.gov/gene/?term=aging). We found that 65 SVs events in 59 genes might be related to skin aging of WXP (Table 2). These SVs were enriched in pathways referred to the formation of skin structures and aging.

**Table 2:** Candidate structural variations related to skin aging.

**No.**	**Chr**	**Start**	**End**	**Length**	**Type**	**Location**	**Symbol**
sv_20920	12	53161161	53161400	239	DEL	EXON=37/37,STRAND=1	CHD3
sv_12600	7	24102759	24102912	153	DUP	EXON=26/44,INTRON=26/43,STRAND=-1	TNXB
sv_10846	6	57942591	57942680	89	DEL	EXON=7/16,INTRON=6/15,STRAND=-1	PPP2R1A
sv_03787	2	48671743	48672091	348	DEL	EXON=7/22,INTRON=7/21,STRAND=1	EIF4G2
sv_03788	2	48672667	48672980	313	DEL	EXON=10/22,INTRON=10/21,STRAND=1	EIF4G2
sv_03789	2	48673120	48673212	92	DEL	EXON=11/22,INTRON=11/21,STRAND=1	EIF4G2
sv_03790	2	48675413	48675496	83	DEL	EXON=16/22,INTRON=16/21,STRAND=1	EIF4G2
sv_07880	4	93902799	93902892	93	DEL	EXON=11/15,INTRON=10/14,STRAND=-1	LMNA
sv_06002	3	76867706	76867956	250	DEL	EXON=6/7,INTRON=6/6,STRAND=1	RAB1A
sv_14248	8	20162216	20162373	157	DEL	EXON=11/11,INTRON=10/10,STRAND=1	RBPJ
sv_24885	14	113163523	113163523	63	INS	EXON=10/14,STRAND=1	NOLC1
sv_07254	4	36223670	36223968	298	DEL	EXON=11/18,INTRON=11/17,STRAND=1	PABPC1
sv_07255	4	36224682	36224763	81	DEL	EXON=13/18,INTRON=13/17,STRAND=1	PABPC1
sv_08996	5	33175088	33175088	88	INS	EXON=19/19,STRAND=1	MDM2
sv_20346	12	17659189	17659247	58	DEL	INTRON=1/4,STRAND=-1	WNT3
sv_01051	1	74645288	74645365	77	DEL	INTRON=2/3,STRAND=1	FOXO3
sv_07988	4	103498496	103498560	64	DEL	INTRON=3/7,STRAND=1	VTCN1
sv_05472	3	27283820	27284082	262	DEL	INTRON=3/11,STRAND=-1	XYLT1
sv_13380	7	89601504	89602362	858	DEL	INTRON=8/12,STRAND=1	FUT8
sv_20057	12	1754287	1754351	64	DEL	INTRON=11/31,STRAND=-1	RPTOR
sv_09701	5	79832933	79833069	136	DEL	INTRON=2/2,STRAND=-1	CHST11
sv_17909	10	14546142	14546464	322	DEL	INTRON=2/10,STRAND=1	PSEN2
sv_06478	3	117923399	117923537	138	DUP	INTRON=11/21,STRAND=1	PUM2
sv_15424	8	124327820	124327887	67	DEL	INTRON=1/15,STRAND=1	UNC5C
sv_14140	8	14819672	14819672	57	INS	INTRON=4/39,STRAND=1	SLIT2
sv_00838	1	53380201	53380503	302	DEL	INTRON=18/26,STRAND=-1	CEP162
sv_02630	1	246244121	246244330	209	DEL	INTRON=2/50,STRAND=-1	ABCA1
sv_22719	13	137104024	137104685	661	DEL	INTRON=12/20,STRAND=1	ADCY5
sv_03472	2	25622346	25622540	194	DEL	INTRON=1/10,STRAND=1	SLC1A2
sv_16374	9	39266560	39266881	321	DEL	INTRON=4/9,STRAND=-1	BTG4
sv_12463	7	14235194	14235332	138	DEL	INTRON=11/16,STRAND=1	RNF144B
sv_12468	7	14574297	14574588	291	DEL	INTRON=16/16,STRAND=1	RNF144B
sv_20094	12	5242599	5242945	346	DEL	INTRON=1/19,STRAND=1	RNF157
sv_02955	1	268335021	268335100	79	DEL	INTRON=1/13,STRAND=-1	ENG
sv_21044	12	59897867	59897867	51	INS	INTRON=1/10,STRAND=1	ALDH3A1
sv_13020	7	53755609	53755689	80	DUP	INTRON=20/23,STRAND=-1	ALDH1L1
sv_11808	6	149630210	149630516	306	DEL	INTRON=9/11,STRAND=-1	ATG4C
sv_27208	15	133594044	133594148	104	DEL	INTRON=13/26,STRAND=1	INPP5D
sv_14236	8	19500083	19500145	62	DEL	INTRON=6/12,STRAND=1	SLC34A2
sv_12131	6	166363129	166363766	637	DEL	INTRON=2/10,STRAND=1	EIF2B3
sv_22679	13	133322069	133322328	259	DEL	INTRON=9/21,STRAND=-1	PAK2
sv_03313	2	16611778	16612068	290	DEL	INTRON=1/10,STRAND=-1	CRY2
sv_11779	6	148337406	148337584	178	DUP	INTRON=7/8,STRAND=-1	ROR1
sv_11783	6	148486408	148486821	413	DEL	INTRON=2/8,STRAND=-1	ROR1
sv_10737	6	44037692	44037873	181	DEL	INTRON=13/29,STRAND=1	GPI
sv_01505	1	119812278	119812356	78	DEL	INTRON=1/12,STRAND=1	LEO1
sv_00508	1	29660305	29660787	482	DEL	INTRON=1/13,STRAND=1	SGK1
sv_29831	18	5418190	5419615	1425	DEL	INTRON=2/9,STRAND=-1	GALNTL5
sv_29632	17	52827801	52828714	913	DUP	INTRON=3/11,STRAND=-1	NFATC2
sv_25217	14	134421991	134422184	193	DEL	INTRON=3/11,STRAND=-1	CTBP2
sv_13418	7	93027107	93027168	61	DEL	INTRON=11/15,STRAND=1	GALNT16
sv_29956	18	13806603	13806603	58	INS	INTRON=1/42,STRAND=-1	NUP205
sv_28139	16	28242760	28242907	147	DEL	INTRON=19/23,STRAND=1	NNT
sv_08616	5	9349445	9349445	65	INS	INTRON=4/8,STRAND=-1	TOMM22
sv_27216	15	134115973	134116307	334	DEL	INTRON=4/8,STRAND=-1	HJURP
sv_22572	13	121939152	121939152	59	INS	INTRON=3/29,STRAND=-1	ABCC5
sv_02568	1	241731580	241731635	55	DEL	INTRON=3/6,STRAND=1	STX17
sv_29840	18	6193747	6194358	611	DEL	INTRON=1/15,STRAND=-1	ABCB8
sv_13477	7	97732176	97732450	274	DEL	INTRON=3/3,STRAND=-1	NPC2
sv_22791	13	146185493	146185553	60	DEL	INTRON=2/15,STRAND=-1	ATP6V1A
sv_24925	14	115794719	115795323	604	DEL	INTRON=2/28,STRAND=1	SORCS3
sv_20679	12	39646645	39646954	309	DEL	DISTANCE=1644,STRAND=1	CCL5
sv_04865	2	143537750	143538086	336	DEL	DISTANCE=2769,STRAND=1	RNF14
sv_04626	2	129802694	129802969	275	DEL	DISTANCE=4352,STRAND=1	LMNB1
sv_29412	17	38646660	38646795	135	DEL	DISTANCE=2728,STRAND=-1	EIF6

### Structural variation validation

To validate the SVs deduced from the deep sequencing data, 38 SVs from 36 genes were randomly selected for validation by PCR analysis. The PCR products were then sequenced by the Sanger method to define the precise breakpoint of SVs. These confirmed SVs contained 36 deletions, 1 insertion, and 1 inversion. Total of 195 pig samples including 43 Xiang pig with wrinkle skin, 78 Xiang pig with normal skin, and 74 Large White pigs were examined by PCR methods. The results showed that 38 SVs and 32 SVs had been detected in genomes from Xiang pigs and Large White pigs, respectively. The bands of agarose gel electrophoresis corresponding with the predicted SV events could be detected in the PCR products (Supplementary Figure 1), and further testified by Sanger sequencing. Supplementary Table 9 showed the polymorphism of thirty-eight SVs in 195 pigs. Most of these SVs were genotyped at high frequency (≥20%) in wrinkle skin samples. The allele frequency of 15 SVs presented a significant difference between the three groups.

Additionally, we used RNA-seq data obtained from the wrinkle skin and the normal skin samples from six Xiang pigs which was the same as the DNA sequencing to investigate the impact of SVs on transcription. For this, we focused on differences in expression levels and alternative splicing events of those genes with SVs specifically presented in wrinkle skin group. Of those 481 protein genes, 428 genes were expressed in skin samples. 26 differentially expressed genes were identified between the wrinkle skin and normal skin samples. Overall, we observed that 65% genes covered deletions generally decreased the expression (Supplementary Table 10). Five basic types of AS events were detected by rMATS from the RNA-seq data, including A5SS (alternative 5' splice site), A3SS (alternative 3' splice site), SE (skipped exon), RI (Retained intron) and MXE (mutually exclusive exon). We identified 106 differentially alternative splicing events from 84 genes which covered specific SVs from the RNA-seq data. SE was the most prevalent differentially alternative splicing events (n=79), followed by RI (n=16), and MXE (n=10). Most of insertion, deletion or duplication were resulted in aberrant or differential splicing in transcripts (Supplementary Table 11).

**Figure 6:**
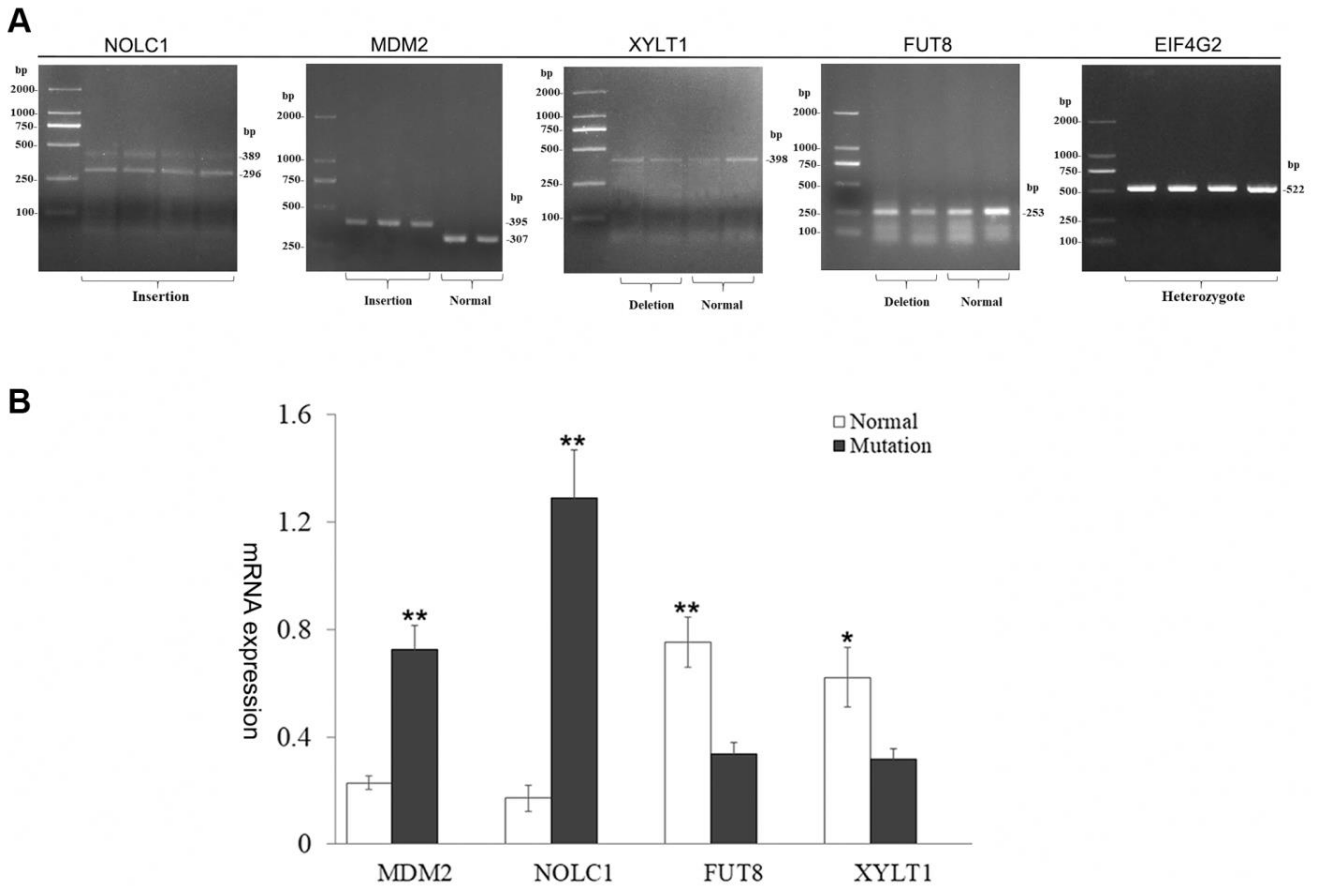
**Characterization of harboring SV gene.** (**A**) Analysis of gene transcripts across SV breakpoints. Two transcripts of *MDM2* and *NOLC1* genes were produced, and the other genes remained unchanged. (**B**) Bar graphs show qRT-PCR evaluation of *MDM2*, *NOLC1*, *FUT8*, *XYLT1* genes. Values are mean ± SEM. t-Test: *, ** indicate *P*< 0.05, *P*< 0.01, respectively.

To further confirm the effect of SV events on gene expression and splicing, we selected an insertion mutation of exon 10 in *NOLC1* gene (sv_24885), two deletion variations (sv_03788, sv_03789) among exon/intron boundaries of *EIF4G2*, one insertion mutations of 88 bp among 3' UTR of *MDM2*, and two intron deletion variants (sv_13380, sv_05472) for RT-PCR and Sanger sequencing validation. We isolated RNAs from the skin samples with same genotypes of the SV and detected the expression patterns (Figure 6A, Supplementary Figure 2). The results showed that 296 bp and 389 bp fragments were detected from the transcripts of *NOLC1*. Sequencing of the RT-PCR product indicated that 296 bp fragment was an abnormal splicing product which retained intron 10. Meanwhile, 395 bp and 307 bp fragments were determined from the transcripts of *MDM2*, respectively. The sequencing revealed that 88 bp fragment was inserted in the 3' UTR of *MDM2.* However, only a single fragment was detected from the transcripts of *EIF4G2*, *FUT8* and *XYLT1.* The sequences of these fragments were in accordance with each other in different genotypes. In addition, we performed qRT-PCR analysis of *NOLC1*, *MDM2*, *FUT8* and *XYLT1* genes. It was found that the expression levels of *MDM2* and *NOCL1* with mutant genotypes were significantly higher than the normal transcripts, while the intronic deletion drove a decrease in transcription of *FUT8* and *XYLT1* genes (Figure 6B).

## DISCUSSION

Under natural conditions, young mammals rarely have systemic skin wrinkles. Skin wrinkles generally appear in adulthood and increase with age. But there are a few special cases in some mammals, such as Chinese Shar-Pei dogs and Birman cats, show systemic skin wrinkles at very young age or at birth. In Xiang pigs, the systemic skin wrinkle phenotype was found in a few of individuals, and appeared in two months after birth and became more and more obvious with age. Originally, the mutated phenotype was observed in a boar and among his offspring, accounting for about 1-3% of the entire populations. The trait of WXP characterizes autosomal recessive heredity, and its genetic mechanism is unknown.

Skin is largely composed of a collagen-rich connective tissue, which provides structural and functional support. Skin aging is associated with structural and functional changes in both of epidermis and dermis. Morphological alterations become apparent in epidermis, including structural changes within the basal membrane and a decrease in cell proliferation [17]. Dermal collagen and elastic fibrils of aging skin are fragmented and disorganized, which impairs the structural integrity and mechanical properties of the skin [18]. In our study, the observation of histological structure indicated that the wrinkle skin of Xiang pig was abnormally thicken, which is similar to aging skins. The epidermal layer proliferated and its thickness was not uniform in skin of WXP. Epidermal hyperplasia hinders the excretory function of skin, which is prone to parasites infection and inflammation in sunken skin. At the same time, we observed that the fibrous structure of the dermis layer was disordered with fiber breakage and decrease of the fiber bundle. Furthermore, subcutaneous fat layer became thinner. Our observation suggested that the aging probably was the cause of the skin wrinkling in Xiang pig.

To uncover molecular mechanism of the skin wrinkle in Xiang pigs, we performed genome resequencing by NGS technology and identified 32,308 SVs from genomes of Xiang pigs and European pigs. Among the identified SVs, 965 SVs were screened from the genome of Xiang pig with wrinkle skin. After annotation, the 965 specific SVs covered 553 Ensembl genes, including protein-coding genes, pseudogenes, lncRNA and snRNA genes. The protein-coding genes were enriched in several KEGG pathways related to skin aging, including RNA transport, Notch signaling pathway, Wnt signaling pathway, mTOR signaling pathway. It indicated that these SV events might mediate the skin phenotypic difference between WXP and XP. RNA transport from the nucleus to the cytoplasm is fundamental for gene expression. In the RNA transport pathway, nuclear pore complexes (NPCs) and translation initiation factors (eIFs) were affected by SV. Particularly, missense mutations in some eIFs induce aging [19]. Notch signaling is directly involved in differentiation in the skin, and impacts on inflammatory processes in the skin [20]. The Wnt signaling pathway is significantly down-regulated in elderly skin [21]. The nuclear lamina was one of significantly enriched GO term. Mutations in the nuclear lamina genes lead to the loss of its function, resulted in a wide range of human degenerative and premature aging diseases, such as Hutchinson–Gilford progeria syndrome (HGPS), where the skin shows premature aging [22]. The wrinkled Xiang pigs appeared skin aging at 2 months old, which may be related to this SV in nuclear lamina. At the same time, changes in nuclear lamina also affect genomic stability, and the genomic instability is known to be one of hallmarks of aging [23]. Nuclear lamina is closely related to structure and function of nuclear membrane, chromatin and nuclear pore complex. In addition, many involved genes have been found to response to external biotic stimulus and immune system regulation. The skin of wrinkled Xiang pigs is rubefaction, which is manifested as low-grade inflammation. This rubefaction is reported to link the immune system: known as inflammaging or inflamm-ageing, seems to be the common biological factor responsible for the decline and the onset of disease in the elderly [24]. Aging cause disorders of multiple components of the immune system, such as increased sensitivity to infections [25]. Our results suggested that the skin wrinkling of Xiang pig was accompanied by changes in many aspects, such as blocked of some RNA transport and translation process, unstable of nuclear lamina, imbalance of the immune system.

In our study, we finally found that 65 SVs in 59 protein genes were probably concerned in the wrinkled skin of Xiang pig. We mapped these genes to NCBI gene database (https://www.ncbi.nlm.nih.gov/gene/?term=aging) and found that 22 genes have been reported to be related to aging. Among them, 13 SVs in nine genes (*CHD3, NOLC, PPP2R1A, EIF4G2, LMNA, TNXB, RAB1A, RBPJ, PABPC1*) could cause the variations of the coding sequences and intron, which resulted in the loss of stop codon or frameshift mutation and aberrant alternative splicing. For example, the deletion of 239 bp among exon 38 and 3'UTR made the *CHD3* loss of 40 coding codons and stop codon, which resulted in truncated polypeptide. CHD3 (chromodomain helicase DNA binding protein 3) is one of NURD components (a ubiquitous chromatin remodeling complex). NURD has been implicated in transcriptional repression at specific promoters and been shown to associate with pericentromeric heterochromatin [26]. Low expression of the protein or loss of the activity of several NURD components including HDAC1 and CHD3 in normal cells are sufficient to recapitulate age-dependent chromatin defects [27]. Our results suggested that the mutant gene might cause CHD3 dysfunctions and age-dependent chromatin defects in Xiang pig cells. An 89 bp deletion was found among intron 6 and exon 7 of *PPP2R1A* gene in WXP, which resulted in frameshift mutation and early termination. PPP2R1A (protein phosphatase 2 scaffold subunit A alpha) is a scaffolding subunit of PP2A (protein phosphatase 2A). PP2A regulates a variety of cellular functions, including DNA replication, transcription, translation, cell apoptosis and so on. Moreover, PP2A is predominantly regarded as a tumor suppressor. Mutations in PPP2R1A frequently occur in cancer, such as lung, breast, and melanoma [28]. *PPP2R1A* is associated with Alzheimer's disease (AD) and specific cognitive domains, and its mRNA and protein levels are elevated in the patient’s brain [29]. Therefore, *PPP2R1A* is a potential target related to the skin aging process of pig. EIF4G2 is one of the components of eukaryotic initiation factor 4F (EIF4F). The deletions variations among exons/intron 7, 10, 11, and 16 of *EIF4G2* were potential abnormal replicating variables. We examined the impact of two SVs in exons/introns on *EIF4G2* mRNA splicing (Figure 6). Sequencing showed that it did not violate the normal processing of *EIF4G2* mRNA. However, the deletion of introns 10 and 11 may affect gene expression. More and more evidences show that the destruction of the translation mechanism greatly promotes the development and progression of cancer [30]. Down-regulation of *EIF4G2* expression will decrease translation and cell proliferation and induce cellular senescence [31]. The *LMNA* gene encodes nuclear lamins, and gives rise to lamin A and lamin C through alternative splicing. Mutations in *LMNA* cause multiple degenerative disorders, such as HGPS, caused by a point mutation in the 5'-terminus site of exon 11 [32]. The product of this abnormal splicing transcript, defined as progerin, is mainly found in skin fibroblasts and undifferentiated keratinocytes [33]. Remarkably, we found the deletion of 93 bp in intron 10 and 11 exon of *LMNA* gene, and the first two bases of exon 11 were ablated, which resulted in frameshift mutations of the gene and a short peptide of 478 aa. *LMNA* gene mutations elicit genomic instability and limit cellular proliferative capacity [34]. In peoples carrying the mutation, a severe premature senility occurs during childhood [5]. In addition to being a nuclear localization signal binding protein, the nucleolus and coiled-body phosphoprotein 1 (NOLC1) can also be used as a partner to shuttle between the nucleolus and the cytoplasm. Overexpression of *NOLC1* induced cell cycle arrest and apoptosis by disturbing the organization of nucleolus [35]. But when *NOLC1* expression is down-regulated under the interference of *shNOLC*, the expression of *MDM2* proto-oncogene is inhibited while the expression of the apoptosis-related genes (such as *TNF-α*) were up-regulated in nasopharyngeal carcinoma (NPC) cells [36]. Our study found a 63 bp insertion in exon 10 of *NOLC1* gene of WXP. This resulted in the retention of intron 10 of *NOLC1* gene and the addition of 11 amino acids to the encoded protein. And the expression of mutant genes was significantly higher than that of normal genes. When the expression of *NOLC1* was up-regulated, it may promote the excessive proliferation of epidermal cells and form a thicker epidermis. Moreover, we found that the 153 bp duplication of intron 26 and exon 26 in *TNXB* gene caused frameshift mutation and truncated or aberrant splicing transcripts. Furthermore, the duplication of 153 bp in exon 26 of *TNXB* resulted in truncated peptide (2823 aa). *TNXB* encode the large extracellular matrix glycoprotein tenascin XB. Tenascin XB is reported to regulate collagen deposition by dermal fibroblasts [37]. In human, variants in *TNXB* cause a rare monogenic autosomal recessive subtype of Ehlers-Danlos syndromes (EDSs) with hyperextensible and fragile skin [38].

Furthermore, other 46 SVs among 44 genes of Xiang pig mainly caused intron variant. Introns are those sequences which are transcribed and subsequently excised from the primary transcript by splicing to produce the mature RNA. Pre-mRNA splicing depends on the recognition of splice acceptor / donor, exon-intron boundaries, and splicing regulatory sequences. The regulatory elements include donor / acceptor sites, exon and intron splicing enhancers (ESE and ISE) and exon and intron splicing silencers (ESS and ISS). Specific splicing activator and repressors (trans-acting elements) correctly recognize these elements and help to perform the splicing process appropriately. Lose or insertion of the sequence containing these elements may lead to incorrect recognition of exons and introns, and can cause the production of abnormal transcripts of mutated genes, which are the main reason of many diseases [39, 40]. And deregulation of pre-mRNA splicing is related to cellular senescence and the aging phenotype [41]. In our study, by using the 46 SV sequences from the introns, we applied for identification of splicing regulatory sequences by software RegRNA 2.0 online. We found that 42 SVs contained one or more regulatory elements, including ESE, ISE, ESS, MBE (Musashi binding element), and PAS (polyadenylation signal). The splice-site mutations of some genes in WXP may affect its senescence phenotype, such as *XYLT1* and *FUT8*. A 262 bp deletion was found from intron 3 of *XYLT1*, which included ESE, SXL binding site, C-to-U RNA editing sites. Xylosyltransferase 1 (XYLT1) catalyzes the first step of adding glycosaminoglycan (GAG) chains to the proteoglycan core protein. Glycosylation modification is essential for normal proteoglycan (PG) function. Mutations in *XYLT1* cause skeletal dysplasia and severe growth retardation [42]. In cultured fibroblasts, UV-caused downregulation of XYLTs results incomplete glycosylation and secretion of protein [43]. The 262 bp deletion fragment in *XYLT1* gene contained 14 trans-acting factor binding sites, which bind to SRp40, SC35 and Sxl proteins (Supplementary Figure 3). Studies have shown that the destruction of ESE lead to exon skipping and expresses low levels of functional protein [44]. In WXP, the expression of homozygous deletion type of *XYLT1* in the skin was significantly lower than that of normal genotype (*P* < 0.05). Loss of ESE in intron 3 might down-regulate the transcription of *XYLT1*. When the expression of *XYLT1* is insufficient, intact form of PG protein and core protein form were reduced, which affects the structure and space filling of skin. And 858 bp deletion was found from intron 8 of *FUT8*, which contained acceptor, ESE, ESS, Polyadenylation sites, MBE, PAS, GU-rich destabilization elements (Supplementary Figure 3). The core protein requires glycosylation to have a function. For example, TGFβ1 lacking the core fucosylation causes a significant imbalance in TGFβ1 receptor activation and signal transduction, resulting in an increase in matrix metalloproteinases (MMPs) [45]. The main molecular mechanism of the skin aging process is attributed to the loss of mature collagen and increased expression of MMPs [46]. Studies have demonstrated that glycosaminoglycans are reduced in aging skin and core proteoglycans are damaged [47]. Point mutations at ESE or ESS can cause aberrant splicing such as exon skipping or intron retention, and produce abnormal transcripts [48]. Loss of ESE, ESS in intron 8 of *FUT8* gene of WXP disrupted splicing regulatory sequences, created probably new ones, or activated the cryptic ones. Alternative polyadenylation sites will produce different C-terminal of protein isoforms, called the coding region-APA (CR-APA) [49]. It is reported that intron polyadenylation of the *Pdgfra* gene leads to increased expression of shorter transcription variants with truncated kinase domains, thereby reducing tissue fibrosis of muscles [50]. Lack of polyadenylation in intron 8 probably resulted in the loss of truncated transcripts of *FUT8* gene. The Musashi binding element inhibits mRNA translation by binding to the Musashi protein family and competing with eIF4G [51]. When the binding site is missing, it will promote translation. The loss of splicing regulatory elements in intron 8 was estimated to be related to *FUT8* gene expression. In fact, the expression of *FUT8* in homozygous deletion mutant samples was much lower than that of normal type of Xiang pig. Insufficient expression of glycosyltransferase will cause glycosylation defects, affect the normal function of proteoglycan, and eventually lead to structural disorder of the extracellular matrix (ECM). Glycosylation defects was likely to a potential factor for skin aging of WXP.

Moreover, deletions were detected in the upstream of four genes (*LMNB1*, *CCL5*, *RNF14*, *EIF6*). After analysis of the deletion sequences, it was found that they all contained transcription factor binding sites. Transcription factors (TFs) are a family of DNA-binding proteins and recognized as the master regulators of gene expression. Evidence has shown that deletion of the conserved DNA binding site of transcription factor upstream of the gene, affects the transcription activity of the promoter and cause the decrease of gene expression [52]. Notably, the four genes are more or less related with skin aging [53–56]. Finally, the insertion of 88 bp among 3' UTR made the elongated 3' tail of *MDM2* gene. Mdm2 is the key negative regulator of the tumour suppressor p53 and as a chromatin modifier [57]. It's reported that activation of endogenous p53 by ablation of Mdm2 can induce accelerated aging phenotypes in mice skin [58]. The 88 bp insertion mutation in the *MDM2* gene might affect its localization and translation efficiency. Studies in human cell lines show that the lengths of 3' UTRs differentially regulate the localization of membrane proteins [59]. And the 3' UTR maintains the proper stability of mRNA and ensure normal mRNA nuclear export and translation efficiency [60]. In our work, the expression level of the insertion mutant transcript of *MDM2* gene was higher than that of normal. Elevated *MDM2* expression is exacerbated chromosome instability in aging mice [61]. This will be a new direction for our in-depth research on the skin wrinkle of Xiang pigs.

In summary, we reported a new skin phenotype in Xiang pigs, which is characterized by systemic wrinkles, thickened skin and wispy hair. We sequenced the genome of the phenotypic variant individuals of WXPs and comparison with data from XP and EUP, and identified 965 SVs specific in WXP, covering 514 protein-coding genes. According to gene function and enrichment analysis, we found that 65 candidate SVs in 59 protein genes were probably related with the systemic wrinkle of WXP. The insertion/deletion and duplication variations in these genes resulted in the loss of stop codon or frameshift mutations, and aberrant alternative splicing of transcripts. These genes are involved in cell lamin filament, intermediate filament cytoskeleton, supramolecular complex, cell differentiation and regulation of macromolecule metabolic process etc. Our study suggested that the loss of function or aberrant expression of these genes lead to structural disorder of nuclear and the extracellular matrix (ECM) in skin cells of Xiang pig, which probably was the genetic mechanisms for the mutant phenotype of Xiang pigs with systemic wrinkle.

## MATERIALS AND METHODS

### Animal ethics and collection

All animal procedures were approved by Guizhou University Subcommittee of Experimental Animal Ethics (EAE-GIU-2020-E015) and were conducted the rules of animal experimental ethics. Fourteen Guizhou pigs were used for resequencing. Normal Xiang pig (XP, n = 7) and Xiang pig with systemic wrinkle (WXP, n = 7) were sampled from Dashandi, Qingzhen city. Ear tissue or blood samples were taken according to standard procedures. The age and farm coordinates of the 14 individuals was shown in Supplementary Table 12.

### Histological examination

The skins of three WXPs and three XPs were used for histological examination, about one years old. The skin above the longissimus dorsi muscle were dissected from WXPs and XPs and then fixed in 4% paraformaldehyde. The longitudinal histological sections derived from skin were stained with hematoxyline and eosine, as well as with the Masson trichrome method to visualize collagen (blue) as previously described [62]. Similarly, taken the skin, removed the epidermis to expose the dermis layer and cut to the appropriate size, placed it in 3% glutaraldehyde fixative solution for initial fixation for 20 h, rinsed 3 times with 0.1 mol·L-1 phosphate buffer, used different volume fractions of ethanol-water solution (10%, 30%, 50%, 75%, 100%) dehydration, each gradient dehydration 15 ~ 20 min, pure ethanol dehydration twice, gradient excessive to acetone, freeze-drying, used ions sputter gold coating to prepare skin samples, observed under a scanning electron microscope.

### DNA extraction, libraries construction and sequencing

The genomic DNA was extracted from the sample and conduct quality testing. The qualified DNA will be used for subsequent sequencing. Each sample was constructed by paired-end library, and the sequencing platform was Illumina HiSeq2500 (Illumina, USA). Sscrofa 11.1 (ftp://ftp.ensembl.org/pub/release-90/fasta/sus_scrofa/dna/) of pig genome sequence was used as reference. The NGS QC toolkit and BWA software with default parameters were adopted to low quality control and clean reads mapping to pig reference genome sequence, respectively. The compared SAM format files will be converted to BAM format by SAMtools.

### Identification of SVs

Pindel [63] and SoftSV software [64] were performed bioinformatics detection of genomic variation in 14 BAM files. Both programs were used default parameters. As Pindel is not appropriate for the calling of translocation and inverted translocation, we only detected SV types of deletion (DEL), tandem repeat (DUP), inverse (INV), insertion (INS). Two conditions were used to control the data quality. First, paired-ends appear at least three times in short read. Second, the SVs were called by both softwares. If two SVs were belonging to the same mutation type on the same chromosome, and overlapped more than 25 bp will be merged into one SV. In addition, to eliminate the influence of gender on SV detection, data from chromosome Y was removed.

To screen the specific genomic structures of WXP, we downloaded the resequencing data of 21 European pigs (EUP) from the public SRA database (https://www.ncbi.nlm.nih.gov/sra/?term=pig) (Supplementary Table 2), including Large White (LW), Landrace (LA) and Duroc (DU). The same method was used to detect EUP confident SVs. We merge SVs between different individuals according to the principle of SVmerge [65]. And SVs detected from two or more individuals were retained to the final call set.

### SV annotation

The SVs were annotated using the Ensembl Variant Effect Predictor tool (http://asia.ensembl.org/Multi/Tools/VEP?db=core). Variant annotations were divided into two types, as high (destructive effects on the protein) or modifier (non-destructive variant), which can be used for genetic analysis of observed phenotypic differences. Then, we used the KOBAS 3.0 tool (http://kobas.cbi.pku.edu.cn/) to perform Gene Ontology (GO) enrichment analysis and Kyoto Encyclopedia of Genes and Genomes (KEGG) pathway analysis.

### SV validation

We evaluated the reliability of the data and verified 38 randomly selected SVs using PCR analysis and direct sequencing methods. Considering the influence of SV on gene transcription and expression, we selected 6 SVs for further analysis. Skin total RNA from different genotypes was isolated using the TRIZOL reagent and reversed transcription. RT-PCR was used to verify whether the transcription near the SV breakpoints were normal. Then, Real-Time polymerase chain reaction performed using iQTM SYBR® Green Supermix (Bio-Rad, CA, USA) in an iQ5 Multicolour Real-time PCR Detection System (Bio-Rad). Experiments were performed in triplicate and expression values were normalized to *GAPDH* levels using the following formula: 2^− (ΔCT)^. Results were presented as the mean ± SEM (standard error of mean). Statistical analyses were performed with SPSS software (IBM, SPSS Statistics, US. version 23). All primers used were listed in Supplementary Table 13.

RNA from the skin of six Xiang pigs, was used for RNA-seq analyses. RNA-seq was performed on the Illumina Hiseq X10 at the Huada Gene Technology in Shenzhen using the 150 paired-end sequencing protocol. The raw data was filtered using Trimmomatic-0.39 software. STAR_master software was used for sequence alignment, using *Sus scrofa* 11.1 (http://ftp.ensembl.org/pub/release-104/gtf/sus_scrofa/) as the reference genome. Subread 2.0.0 featurecounts was used to count gene expression. The expression of 6 samples were standardized by calculating Counts Per Million (CPM) [66]. The R package DESeq2 was used to analyze the difference in gene expression between XP and WXP skin. And rMATS predicted AS events, including A5SS (alternative 5' splice site), A3SS (alternative 3' splice site), SE (skipped exon), RI (Retained intron) and MXE (mutually exclusive exon).

## Supplementary Material

Supplementary Figures

Supplementary Tables 1-5

Supplementary Table 6

Supplementary Table 7

Supplementary Table 8

Supplementary Table 9

Supplementary Table 10

Supplementary Table 11

Supplementary Tables 12, 13
