# Supplementary material for: Detection of genomic structure variants associated with wrinkled skin in Xiang pig by next generation sequencing
Source: Aging (Albany NY). 2021 Nov 27;13(22):24710–39. doi: 10.18632/aging.203711 (PMC8660620; doi:10.18632/aging.203711)
Supplement: Supplementary Figures [file aging-13-203711-s001.pdf]

SUPPLEMENTARY FIGURES

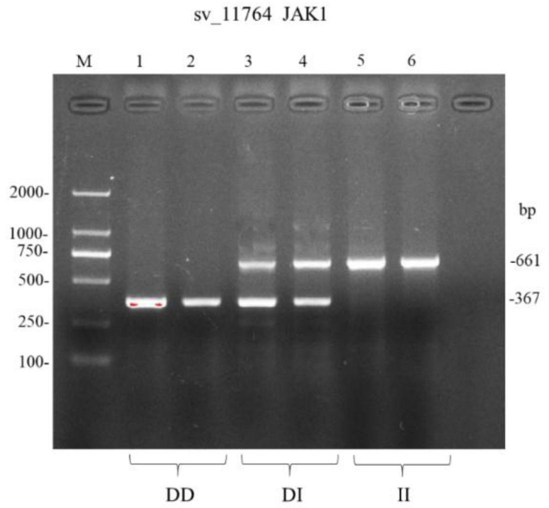

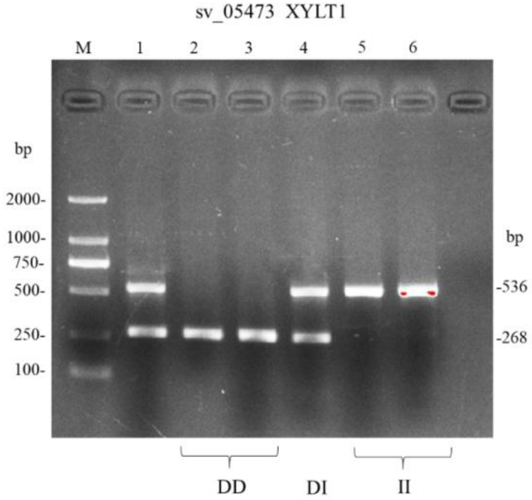

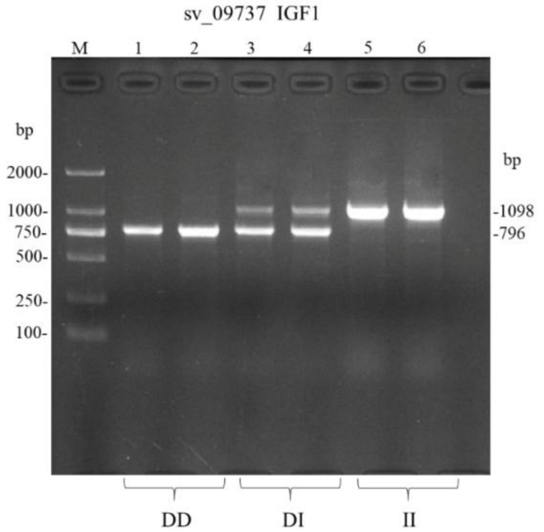

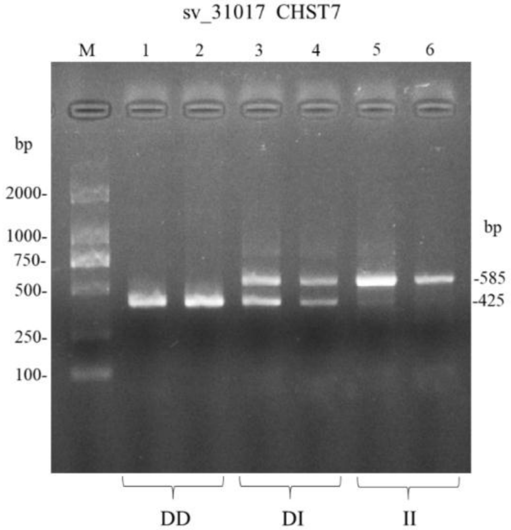

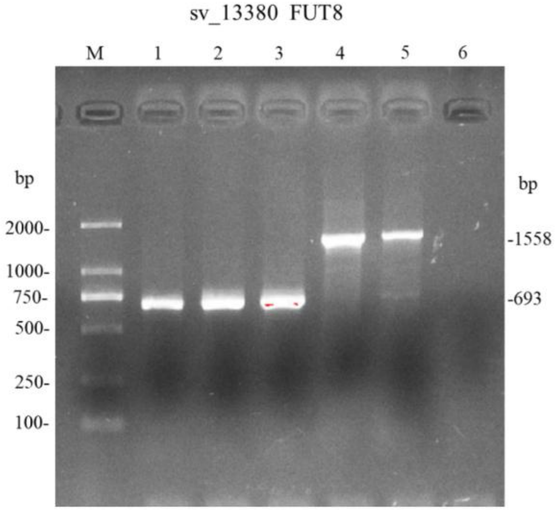

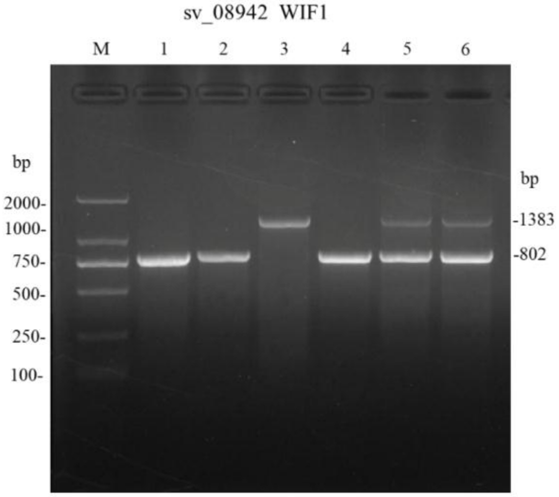

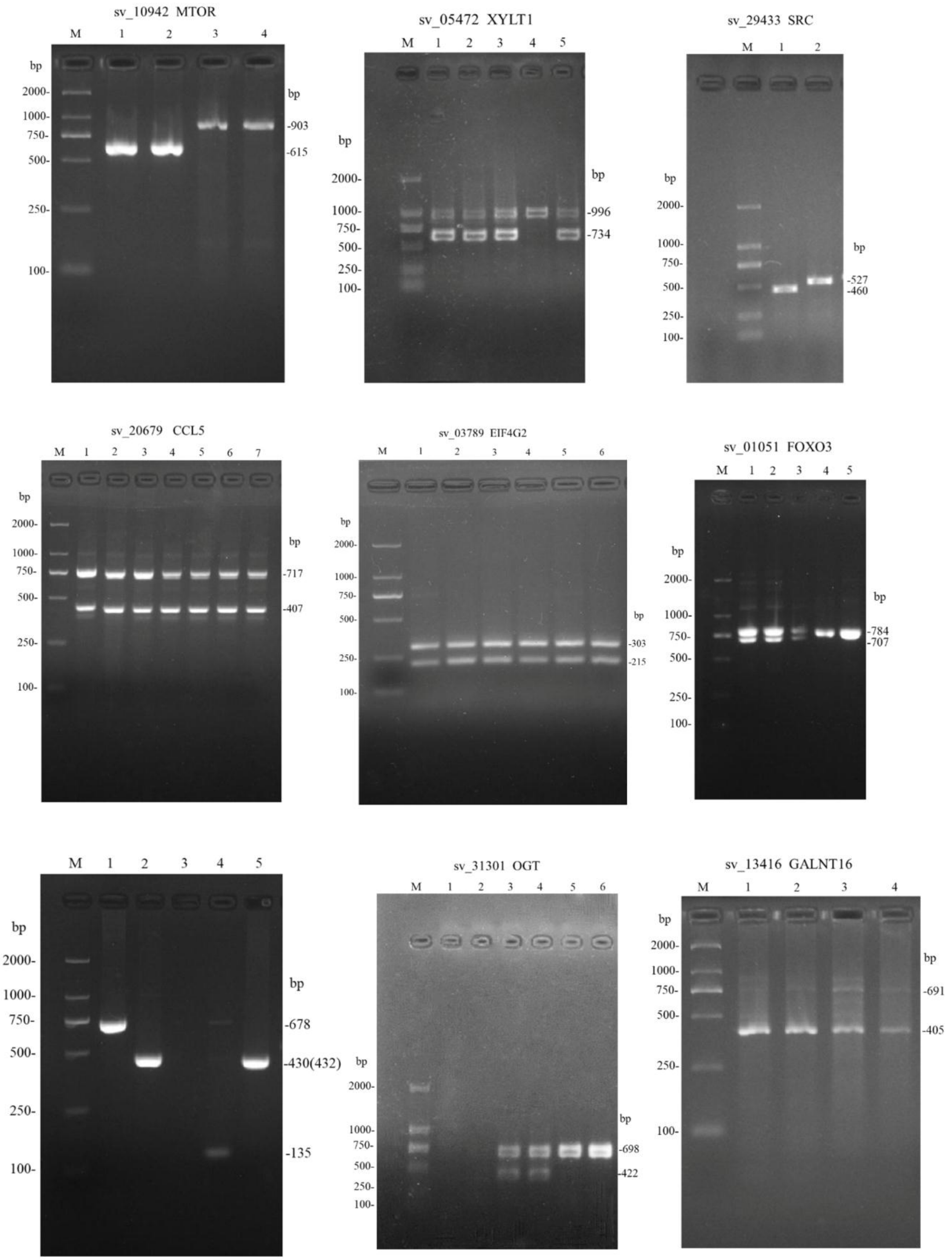

sv_02434 GCNT1: 1(678bp), 2(430bp)

sv_13222 DAD1: 4(135bp), 5(432bp)

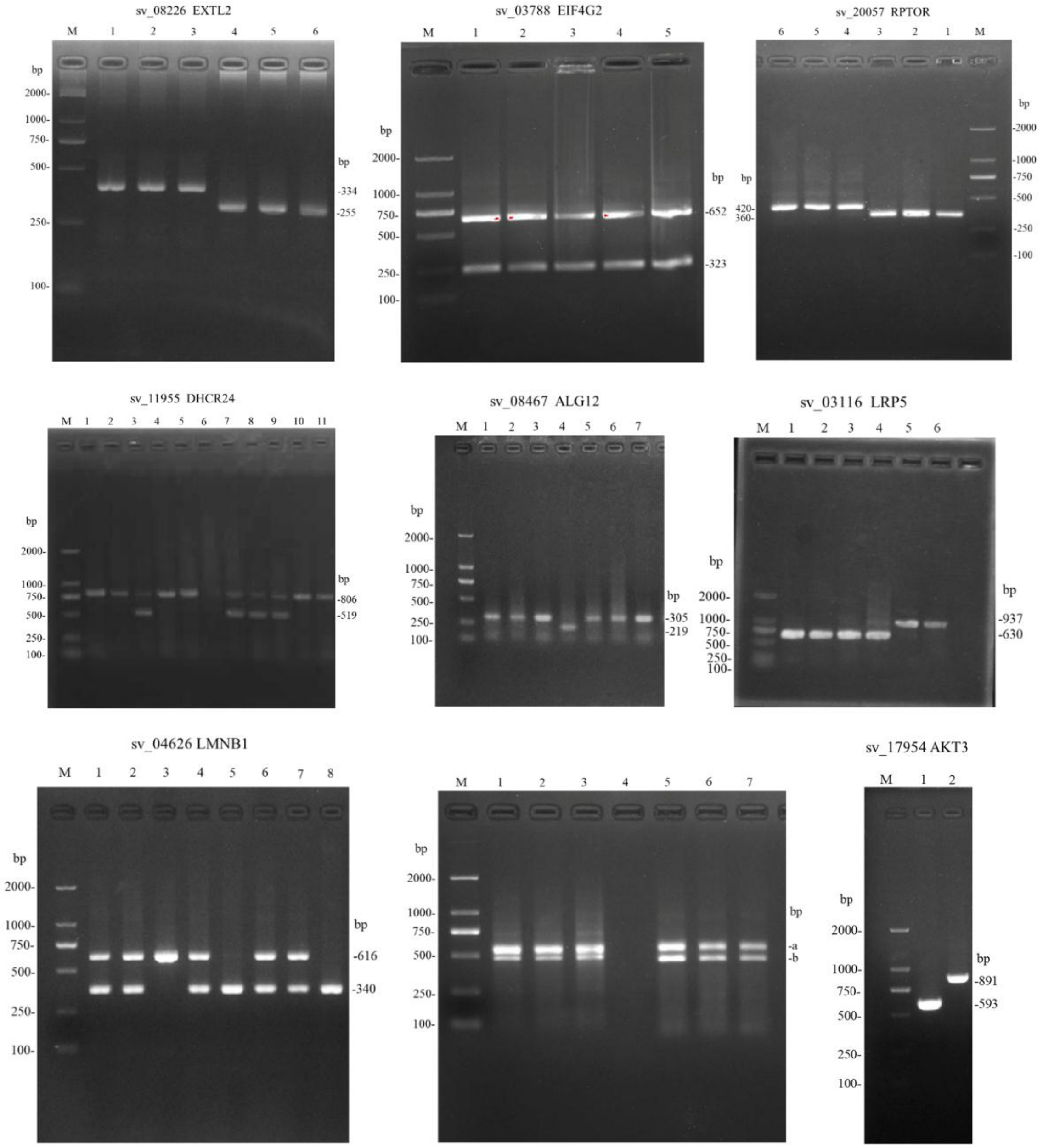

sv_20346 WNT3: 1a(531bp), 1b(472bp)

sv_07988 VTCN1: 5a(520bp), 5b(455bp)

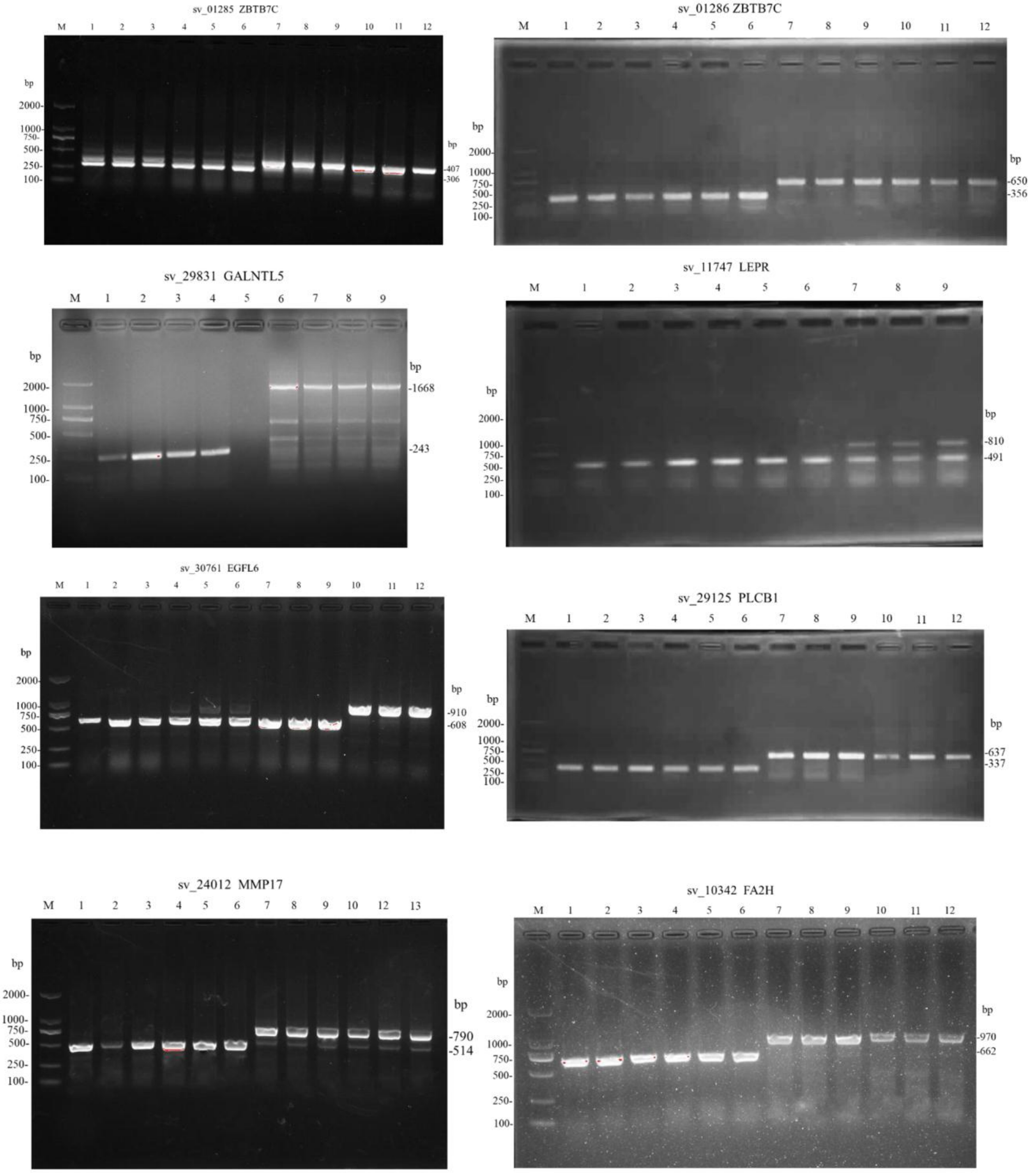
sv_01285 ZBTB7C
M 1 2 3 4 5 6 7 8 9 10 11 12
bp
2000- 1000- 750- 500- 250- 100-
bp -407 -306
sv_01286 ZBTB7C
M 1 2 3 4 5 6 7 8 9 10 11 12
bp
2000- 1000- 750- 500- 250- 100-
bp -650 -356
sv_29831 GALNTL5
M 1 2 3 4 5 6 7 8 9
bp
2000- 1000- 750- 500- 250- 100-
bp -1668 -243
sv_11747 LEPR
M 1 2 3 4 5 6 7 8 9
bp
2000- 1000- 750- 500- 250- 100-
bp -810 -491
sv_30761 EGFL6
M 1 2 3 4 5 6 7 8 9 10 11 12
bp
2000- 1000- 750- 500- 250- 100-
bp -910 -608
sv_29125 PLCB1
M 1 2 3 4 5 6 7 8 9 10 11 12
bp
2000- 1000- 750- 500- 250- 100-
bp -637 -337
sv_24012 MMP17
M 1 2 3 4 5 6 7 8 9 10 12 13
bp
2000- 1000- 750- 500- 250- 100-
bp -790 -514
sv_10342 FA2H
M 1 2 3 4 5 6 7 8 9 10 11 12
bp
2000- 1000- 750- 500- 250- 100-
bp -970 -662

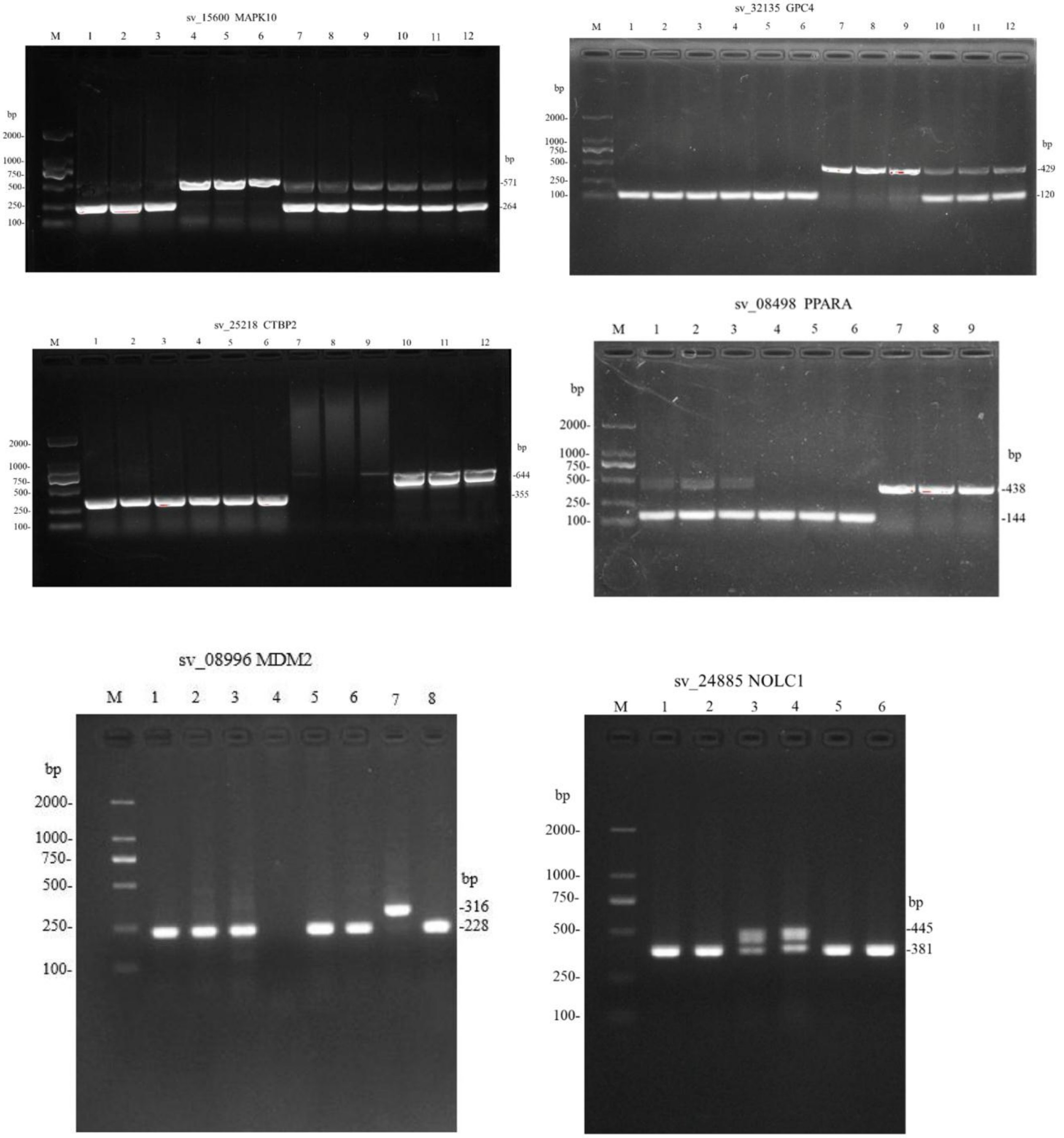
sv_15600 MAPK10
M 1 2 3 4 5 6 7 8 9 10 11 12
bp
2000-
1000-
750-
500-
250-
100-
-571
-264
sv_32135 GPC4
M 1 2 3 4 5 6 7 8 9 10 11 12
bp
2000-
1000-
750-
500-
250-
100-
-429
-120
sv_25218 CTBP2
M 1 2 3 4 5 6 7 8 9 10 11 12
2000-
1000-
750-
500-
250-
100-
bp
-644
-355
sv_08498 PPARA
M 1 2 3 4 5 6 7 8 9
bp
2000-
1000-
750-
500-
250-
100-
-438
-144
sv_08996 MDM2
M 1 2 3 4 5 6 7 8
bp
2000-
1000-
750-
500-
250-
100-
-316
-228
sv_24885 NOLC1
M 1 2 3 4 5 6
bp
2000-
1000-
750-
500-
250-
100-
-445
-381

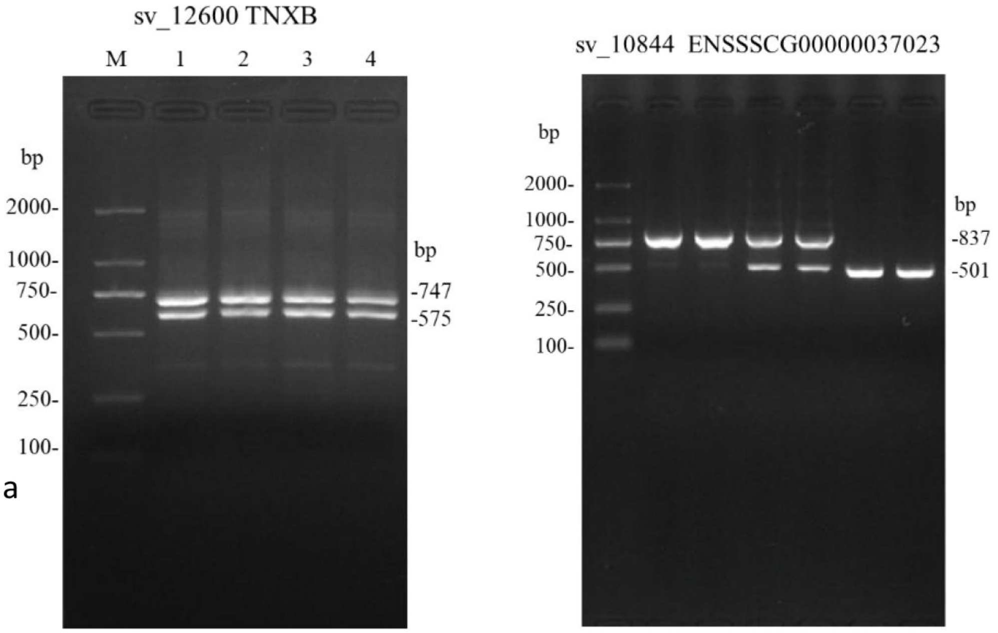

Supplementary Figure 1. Representative gel images for the confirmation of SVs. Note: About 38 SVs really exist in genome. Most genes detected three genotypes, namely normal genotype (II), heterozygous genotype (DI), mutant genotype (DD). See diagram SV_11764.

MDM2

Aberrant transcripts (395 bp)

Sbjct 33174918 TCAGACAAGCAAATAGCCCTCTTTCATAGAAAGACTGAGCACTTTATAGTCAGATGCCTC 33174977

Sbjct 33174978 AGCACTGGGCCCTGAGGGTAGCCAGGTAAGTCTGCGAGAGTCCTTTAGGTACTGTCTCTC 33175037

Sbjct 33175038 AGTTTGCTATAGCCTTGTGGTTCTCATGGATACAAACCCTGTCTCTTTCAAAGCTAGATG 33175097

Sbjct 33175098 TTTTGTAGGCTTGTCTCTTAGGTGCAGGTCTTAAAAGTTGGGGTGCCAGATGTGGGATTC 33175157

Sbjct 33175158 AAACATTTTCTTCTCAGGGA 33175177

AGCTAGATGTTTTGTAGGCTTGTCTCTTAGGTGCAGGTCTTAAAAGTTGGGGTGCCAGATG

TGGGATTCAAACATTTTCTTCTCAGGGA

Sbjct 33175178 GCAGCTTGGAGTTGTGTGTTCCTCCTTGATTGTGGGTTGCTATACCA 33175224

NOLC1

pre-mRNA (389 bp)

Sbjct 113163333 TACCAAAAGCCGCTACTAAAGCAGCTCCAGCAAAGAAGGCAGCAGAGAGCTCTTCAGATA 113163392

Sbjct 113163393 GCTCAGGTAAGGCATATGGAGGCCTTTGGGGCAGTGGGAGCTCCAGGGACTCCCCAGAGT 113163452

Sbjct 113163453 CTAGTTTGGGGAGCCACTCATTCTATTCTTGCTGTCTAGACTCTGACAGTTCTGAGGATG 113163512

Sbjct 113163513 AAGCTCCTGCCAAGCCAGCCAGTGCCACCAAGAATTCAAGTAAGCCAGCTGCCACTCCCA 113163572

Sbjct 113163573 AGCAGTCTATAG 113163584

CTAAGCCAGCCAGTGCCACCAAGAATTCAAGTAAGCCAGCTGCCACTCCCAAGCAGTCTATAG

Sbjct 113163585 CTAAGCCAGCCACAAGTTCCAAGCAGCCTGTAGGCAGTGGCCAGAAGCCTCTGACCAGAA 113163644

Sbjct 113163645 AGGCTGATAGCAGCTCCAGTGAGGAGGAGAGCAGTTCTAGTGAGGACGAGAAGATGAAGA 113163704

Sbjct 113163705 AGACTGTAGCAACGCCT 113163721

Aberrant transcripts (296 bp)

Sbjct 113163333 TACCAAAAGCCGCTACTAAAGCAGCTCCAGCAAAGAAGGCAGCAGAGAGCTCTTCAGATA 113163392

Sbjct 113163393 GCTCAG 113163398

Sbjct 113163492 ACTCTGACAGTTCTGAGGATGAAGCTCCTGCCAAGCCAGCCAGTGCCACCAAGAATTCAA 113163551

Sbjct 113163552 GTAAGCCAGCTGCCACTCCCAAGCAGTCTATAGCTAAGCCAGCCACAAGTTCCAAGCAGC 113163611

Sbjct 113163612 CTGTAGGCAGTGGCCAGAAGCCTCTGACCAGAAAGGCTGATAGCAGCTCCAGTGAGGAGG 113163671

Sbjct 113163672 AGAGCAGTTCTAGTGAGGACGAGAAGATGAAGAAGACTGTAGCAACGCCT 113163721

Supplementary Figure 2. The abnormal transcript information of *MDM2* and *NOLC1* gene. Note: Blue letters represent exons, black letters represent introns. And the red shading and yellow filled sequences denote insertion site and variant fragment, severally.

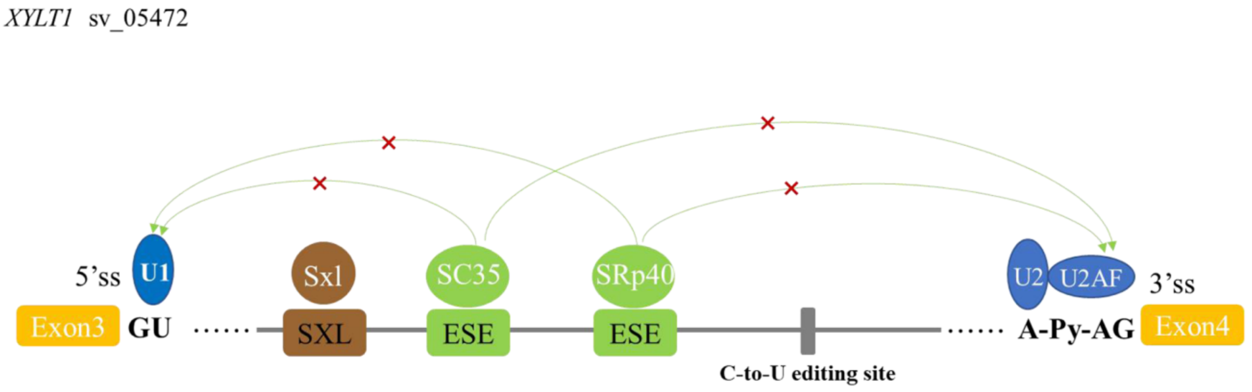

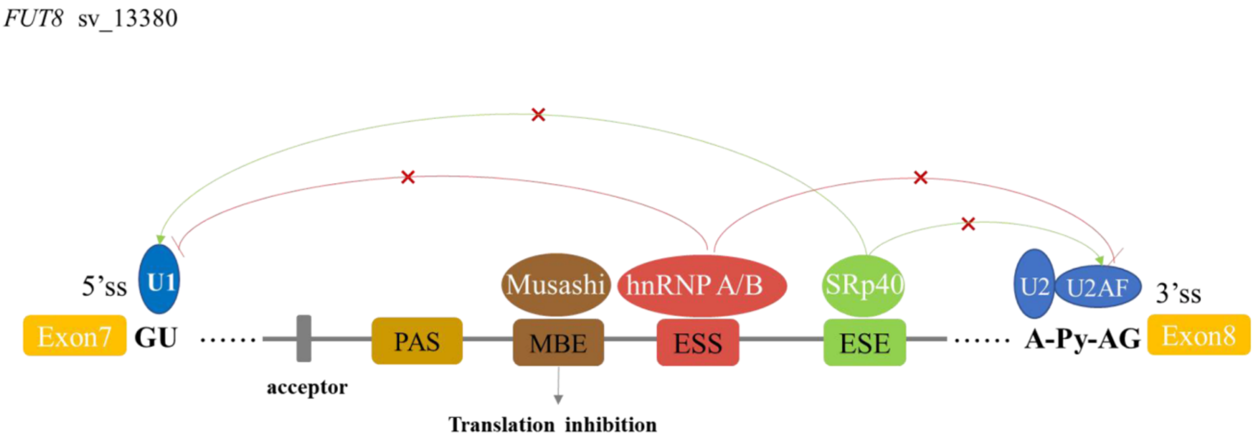

Supplementary Figure 3. Splice sites, regulatory elements and splicing alterations caused by mutations. Note: Intron mutations lead to the loss of splicing elements and may lose the enhancement or inhibition of splicing.
