# Supplementary material for: Detection of genomic structure variants associated with wrinkled skin in Xiang pig by next generation sequencing
Source: Aging (Albany NY). 2021 Nov 27;13(22):24710–39. doi: 10.18632/aging.203711 (PMC8660620; doi:10.18632/aging.203711)
Supplement: Supplementary Tables 1-5 [file aging-13-203711-s002.pdf]

SUPPLEMENTARY TABLES

Supplementary Table 1. Statistics of WXP incidence in recent years.

Years	Number of piglets	Wrinkled pigs	Occurrence rate(%)
2016	480	5	1.04
2017	587	13	2.21
2018	725	22	3.03
2019	832	26	3.13
2020	403	9	2.23

Supplementary Table 2. Skin thickness and subcutaneous fat thickness of Xiang pigs.

	Sample	Month	Skin thickness (mm)	Subcutaneous fat thickness(mm)	Average skin thickness (mm)	Average thickness of subcutaneous fat (mm)
	1	9.5	5	20		
	2	9	4	17		
	3	9	5	16		
	4	9	4	25		
	5	9.5	4	22		
	6	9.5	5	18		
	7	9	5	15		
	8	9	4	20		
	9	9	5	20		
	10	9	3	30		
	11	9	4	20		
	12	9	3	28		
XP	13	9	4	23	4.42±0.71	17.84±6.89
	14	9	5	20		
	15	5.4	3	25		
	16	5.5	4	11		
	17	5.5	5	14		
	18	5.4	4	25		
	19	5.4	4	11		
	20	5.5	4	14		
	21	5.4	3	17		
	22	5.5	5	21		
	23	5.5	4	16		
	24	5.4	4	22		
	25	20	5	44		
	1	18	9.5	4		
	2	24	9	20		
WXP	3	30	7	26	9.10±3.75[A]	15.2±9.65
	4	36	15	20		
	5	5.5	5	6		

[A], and [a]denote $P < 0.01$, $P < 0.05$, respectively, two-tailed t test.

Supplementary Table 3. The epidermal thickness of Xiang pigs.

Populations	Epidermal thickness(n=60, μm)
WXP	212.00±124.24^{A}
XP	104.38±34.29

^{A}, and ^{a}denote *P* < 0.01, *P* < 0.05, respectively, two-tailed t test.

Supplementary Table 4. The information of 21 pig resequencing data downloaded from the NCBI.

Accession	Breed	Individuals	Raw Base(G)	Clean Base(G)	Map Base(G)	Map ratio(%)	Depth(X)
ERR173180	Landrace	LA1	19.00	17.91	17.88	99.83	7.31
ERR173181	Landrace	LA2	28.10	26.71	26.65	99.78	10.90
ERR173182	Landrace	LA3	18.40	17.37	17.26	99.37	7.06
ERR977276	Landrace	LA4	12.26	10.55	10.15	96.21	4.15
ERR977277	Landrace	LA5	17.61	15.90	15.35	96.54	6.28
SRR1581042	Landrace	LA6	29.89	25.87	24.31	93.97	9.94
SRR1581043	Landrace	LA7	32.62	28.61	26.85	93.85	10.98
ERR173198	Large White	LW1	17.60	16.82	16.60	98.69	6.79
ERR173196	Large White	LW2	19.20	18.31	18.21	99.45	7.45
ERR173197	Large White	LW3	17.50	16.52	16.38	99.15	6.70
SRR4341302	Large White	LW4	23.00	22.78	21.85	95.92	8.94
SRR4341326	Large White	LW5	20.60	20.31	19.72	97.10	8.07
SRR3123346	Large White	LW6	35.51	33.64	33.01	98.13	13.50
SRR3123347	Large White	LW7	46.88	44.68	43.63	97.65	17.84
SRR5337426	Duroc	DU1	17.75	17.20	16.68	96.98	6.82
SRR5337627	Duroc	DU2	16.13	15.62	15.22	97.44	6.22
SRR5337652	Duroc	DU3	17.21	16.70	16.25	97.31	6.65
SRR5337661	Duroc	DU4	16.95	16.41	16.06	97.87	6.57
SRR4341281	Duroc	DU5	27.60	26.93	25.60	95.06	10.47
SRR5351767	Duroc	DU6	17.99	17.47	17.06	97.65	6.98
SRR5357804	Duroc	DU7	16.75	16.10	15.68	97.39	6.41

Supplementary Table 5. Counts of raw SVs in pig genomes detected by Pindel and SoftSV programs.

Group	pig	Pindel	SoftSV	Overlap	DEL	DUP	INV	INS
	WXP1	22534	18672	6329	5225	424	25	655
	WXP2	22699	22338	7565	6572	387	25	581
	WXP3	15901	29093	8905	8099	317	29	460
WXP	WXP4	16839	28919	9555	8618	367	32	538
	WXP5	26358	36836	12490	10918	634	64	874
	WXP6	28204	43634	13105	11644	572	35	854
	WXP7	27961	43657	12995	11442	600	30	923
	XP1	21986	29120	12368	11268	307	99	694
	XP2	25251	30300	12915	11465	372	124	954
	XP3	31268	39334	19109	17048	877	192	992
XP	XP4	22106	28521	12066	10946	296	97	727
	XP5	23033	29205	12193	10988	302	97	806
	XP6	31322	39243	19006	16887	886	180	1053
	XP7	22615	39593	12549	11174	515	28	832
	LA1	1972	2290	1280	1170	82	3	25
	LA2	2760	2696	1389	1232	105	8	44
	LA3	1869	2035	970	881	63	2	24
	LA4	4541	9046	2804	2538	224	13	29
	LA5	7784	13642	4926	4427	417	15	67
	LA6	11501	16769	7439	6701	557	20	161
	LA7	12033	17911	8554	7221	1166	23	144
	LW1	1839	2095	1119	1049	40	6	24
	LW2	1942	2175	1197	1103	61	5	28
	LW3	1786	2047	1096	1014	46	5	31
EUP	LW4	11712	15013	7444	6696	511	22	215
	LW5	10142	13403	6523	5895	480	27	121
	LW6	13183	15171	6950	5929	801	14	206
	LW7	15600	18047	8669	7458	933	17	261
	DU1	5905	9021	3459	2986	400	8	65
	DU2	5131	8687	3148	2790	298	6	54
	DU3	5839	9055	3515	3061	388	7	59
	DU4	5597	8831	3361	2937	352	7	65
	DU5	10877	13403	5876	5155	492	42	187
	DU6	6067	9085	3489	2994	411	9	75
	DU7	12112	18389	8436	7456	816	21	143
