# Supplementary material for: Detection of genomic structure variants associated with wrinkled skin in Xiang pig by next generation sequencing
Source: Aging (Albany NY). 2021 Nov 27;13(22):24710–39. doi: 10.18632/aging.203711 (PMC8660620; doi:10.18632/aging.203711)
Supplement: Supplementary Tables 12, 13 [file aging-13-203711-s009.pdf]

Supplementary Table 12. Information of the pig breeds used for resequencing.

Groups	Individual	Sex	Month	Coat color	Coordinates
Normal Xiang pig	XP1	female	7	black	25°16′–26°05 ′ N 108°05 ′ –109°12 ′ E
	XP2	female	10	black	
	XP3	female	13	black	
	XP4	female	7	black	
	XP5	female	13	black	
	XP6	female	7	black	
	XP7	female	20	black	
Xiang pig with wrinkled skin	WXP1	female	27	black	26°25 ′ ~26°56 ′ N 106°07 ′ ~106°32 ′ E
	WXP2	female	27	black	
	WXP3	female	10	black	
	WXP4	female	10	black	
	WXP5	female	18	black	
	WXP6	female	24	black	
	WXP7	female	30	black	

Supplementary Table 13. Primers used for validation by PCR method and used for real-time PCR.

NO.	Chr	Start	End	Primer name	Primer_F (5'--3')	Primer_R (5'--3')	Expected length	Method
sv_01051	1	74645288	74645365	FOXO3	CACAGTGGACAACATCTGGG	GGGAGAGCAGCTGGGAAAAG	784	
sv_01285	1	97940349	97940450	ZBTB7C	GGAAAGGAAGGCAGGGAAG	GTGGTGCAGCAGAAACGAAT	407	
sv_01286	1	98006602	98006896	ZBTB7C	TGCTTGCTCTTGACGAGTTCC	ATCCATCATTGTCGCTGCTGT	650	
sv_02434	1	229459007	229459261	GCNT1	GCAGGCTTGCCAGTGATGA	GCAGCCTGGATCCCTCATT	678	
sv_03116	2	4541120	4541427	LRP5	AGGAGCCATAAGGAGGACGAG	GGTAAGGTTGATGCCCAAACA	937	
sv_05472	3	27283820	27284082	XYLT1-1	CATGCCAAGGGGAAGAATGAG	GGAGCAGCTCAGCAGAGAACC	996	
sv_05473	3	27331301	27331569	XYLT1-2	CCGTTCAGCACACTCACAACC	AGCCACAGCGAGAACTTCACC	536	
sv_08226	4	117421213	117421292	EXTL2	AGGTAGAGGGAGTGGGAAGGAGT	TAGTGGTACCAGGATGACGCAGT	334	
sv_08467	5	715869	715955	ALG12	ACACCACAGCTCACAACAACACT	AGAACTTTGGAGGTGGAAACACA	305	
sv_08498	5	3306982	3307276	PPARA	CCTGGAACGTGCCTTTCATA	GGGGAGTGACTTGGGAAGAG	438	
sv_08942	5	29514283	29515175	WIF1	GGGAACTTTCAGATGTCTCAGG	GGATATGGTCATTTTGGCACTA	1383	
sv_09737	5	81791286	81791588	IGF1	AGGCCCTCGCTTTTGTCTGA	CCCCTGGGTGGTGTAAATGC	1098	
sv_10942	6	71407644	71407932	MTOR	AACAAAGGACCGAAACGCAGA	CAGGTCCCTGAAAGGCAGAGA	903	
sv_11747	6	146886903	146887222	LEPR	GAAACGCAAAGCAAAGGAAAG	TGGGTAACAGTGGTGAGGTGG	810	
sv_11955	6	157511195	157511492	DHCR24	TAGGAGGAGGTGTCGGGTCA	GTGTTGGGGGAGGGATGAAT	806	
sv_13222	7	76426847	76427159	DAD1	CGTTCTCTTTGGAGGTTTTGTG	GAAAGTGGAAGGGCAAGAAAGT	432	
sv_13380	7	89601504	89602362	FUT8	TGGCTTTGCATCTGAAGACC	ACACGTCCCTGAGGCTCTATT	1551	
sv_13416	7	93011572	93011858	GALNT16	CCCTGAGGTTTCTGAGTGGTG	GTGGAATTGGAGCAGCATAGC	691	
sv_15600	8	132650654	132650961	MAPK10	CACTCTGTTCCCATCCGTCCT	AGCCCTCTGCCTGTTTCTTCC	571	
sv_31301	X	57551725	57552001	OGT	CCCCAGAACCGCATCATTTT	CCGCAACTCGAGAAGCAAGA	698	
sv_29831	18	5418190	5419615	GALNTL5	AGATAGTCACTGGGCTGTTCACT	TCTCTCATTGCCTGTGCTGTT	1668	PCR method
sv_24012	14	23487565	23487841	MMP17	GAGGGGCCTGGGCAGTTAGT	AGGCCTCGTTGTGGTTCAGC	790	
sv_25218	14	134463049	134463358	CTBP2	TGCTGACCAGGTAGTGCTTCT	GTTCCCGTTTGGATTTTGTTT	664	
sv_29125	17	17645605	17645905	PLCB1	AGAAAACAGGGCCCAAGAAT	GGCCAAGCAAGAAATGAAAG	637	
sv_29433	17	40526127	40526195	SRC	TTTCTCTGTGCCTTGCTGCTG	GTGGACGCTGCTCTCACCTCT	527	
sv_30761	X	10256511	10256813	EGFL6	CATGCCTCCCTCCTCCTTAG	ATAAACACCAACCACCTCCAT	910	
sv_31017	X	41214172	41214332	CHST7	CACCCCCTGTCCAGAATGT	TTCCGAGGTCAGTCTCCCA	585	
sv_10342	6	12870865	12871173	FA2H	TGGGGTGGGGTGAGAGATACT	TAGGGACGCAGGGAATAAGGA	970	
sv_20057	12	1754287	1754351	RPTOR	CGGGTCTGGAAAATGAGATGC	AGGGACTTTCAGGGCTTGAGG	420	
sv_03788	2	48672667	48672980	EIF4G2-1	TTGCCAGCAAGGATTCGTTT	GTGGGTCCCTATCCATTTTCA	652	
sv_03789	2	48673120	48673212	EIF4G2-2	TGCCACCCAGGATGAAAATG	CCTCCCATCTCCCCAAACTG	303	
sv_20679	12	39646645	39646954	CCL5	CCCTCCACCAGCACTGACA	CGTTCCTGGACTGGAGATTTG	717	
sv_20346	12	17659189	17659247	WNT3	TGGAGAAGGAGTTTGTCGTGG	AGGGGCAAAGAGAGAGGAGG	531	
sv_07988	4	103498496	103498560	VTCN1	GTGGAGAGAGAGAAGAAAATGGG	AGGATCAACAAGGAGGCTCACT	520	
sv_11764	6	147384007	147384301	JAK1	ACACTCATTGAGCCCAGACAGC	GGGTCCCAGGAAGGAAGAAAGT	661	
sv_32135	X	109358785	109359093	GPC4	GAGATCTCTGCGGTCCTCTG	GCAGCCCAGTTGTAAGAAGC	429	
sv_24885	14	113163523	113163523	NOLC1	CAGCAAAGAAGGCAGCAGAGA	TGGCCGTCACCTTGGACTTA	381	
sv_04626	2	129802694	129802969	LMNB1	CTGGCACCTGGTTGAGATTTG	TTCTGCCCTGATTGGAACTGA	616	
sv_08996	5	33175088	33175088	MDM2	CCCTGAGGGTAGCCAGGTAAGTC	ACCCACAATCAAGGAGGAACACA	228	
sv_17954	10	16514855	16515153	AKT3	CCGGGACAGGTGGTTATTCTA	GACTGTTCGCATTTTGATGGA	891	
sv_12600	7	24102759	24102912	TNXB	TTACCAGCAAAGCAAGTCACCC	AGGCCGTTCTCTACCCAGGAG	575	
sv_10844	6	57587714	57588050	sv_10844	TGTTTGTGGGAAAGCCGTTAGT	TCTATGTGGATTGCCAGATGATG	837	
sv_13380	7	89601504	89602362	AS-FUT8	CAAACAGATGGGGCAGGTGA	CCCATCCCCCAGTAGCGTAG	253	
sv_08996	5	33175088	33175088	AS-MDM2	TCAGACAAGCAAATAGCCCTC	TGGTATAGCAACCCACAATCAAG	307	
sv_24885	14	113163523	113163523	AS-NOLC1	TACCAAAAGCCGCTACTAAAGC	AGGCGTTGCTACAGTCTTCTTC	296	Real-Time PCR
sv_05472	3	27283820	27284082	AS-XYLT1	TGATGCCGGAGAAGGTCACTC	GGCGCTGAGGTTGATGAAGAA	398	
sv_03788<br>sv_03789	2<br>2	48672667<br>48673120	48672980<br>48673212	AS-EIF4G2	CCTTGCAGCTGGGATAAGAG	TGCCAGAATGTGTTCCTTGA	522	
				GAPDH	AGGTCGGAGTGAACGGATTTG	ACCATGTAGTGGAGGTCAATGAAG	118	
